# Hurricane risk assessment in a multi-hazard context for Dominica in the Caribbean

**DOI:** 10.1038/s41598-023-47527-5

**Published:** 2023-11-23

**Authors:** Peter Sammonds, Akhtar Alam, Simon Day, Katerina Stavrianaki, Ilan Kelman

**Affiliations:** 1 Institute for Risk and Disaster Reduction (IRDR), University College London (UCL), Gower Street, London, WC1E 6BT UK; 2 Department of Geography and Disaster Management, University of Kashmir, Srinagar, 190006 India; 3 Department of Statistical Science, University College London (UCL), 1-19 Torrington Place, London, WC1E 7HB UK; 4 Institute for Global Health, University College London (UCL), Gower Street, London, WC1E 6BT UK; 5 University of Agder, Kristiansand, Norway

**Keywords:** Natural hazards, Environmental impact, Climate-change adaptation

## Abstract

Hurricanes can trigger widespread landslides and flooding creating compound hazards and multiple risks for vulnerable populations. An example is the island of Dominica in the Caribbean, where the population lives predominantly along the coast close to sea level and is subject to storm surge, with steep topography rising behind, with a propensity for landslides and flash river flooding. The simultaneous occurrence of the multiple hazards amplifies their impacts and couples with physical and social vulnerabilities to threaten lives, livelihoods, and the environment. Neglecting compound hazards underestimates overall risk. Using a whole island macroscale, (level-I) analysis, susceptibility scenarios for hurricanes, triggered landslides, and floods were developed by incorporating physical process parameters. The susceptibilities were combined with vulnerability indicators to map spatial patterns of hurricane multi-risks in Dominica. The analysis adopted a coupled approach involving the frequency ratio (FR), analytic hierarchy process (AHP), and geographic information system (GIS). Detailed hazard modelling was done at selected sites (level-II), incorporating storm surge estimates, landslide runout simulations, and steady flow analysis for floods. High-resolution terrain data and simulation models, the Rapid Mass Movement Simulation (RAMMS) and the hydrologic engineering center’s river analysis system (HEC-RAS), were employed. Ground validation confirmed reasonable agreement between projected and observed scenarios across different spatial scales. Following the United Nations Office for disaster risk reduction (UNDRR) call for the inclusion of local, traditional, and indigenous knowledge, feedback, and expert opinion to improve understanding of disaster risk, 17 interviews with local experts and 4 participatory workshops with residents were conducted, and findings were incorporated into the analysis, so as to gain insights into risk perceptions. The study’s outcomes encompass projections and quantification of hurricane compound hazards, vulnerabilities, accumulated risks, and an understanding of local priorities. These findings will inform decision-making processes for risk mitigation choices and community actions by providing a new framework for multi-hazard risk assessment that is easy to implement in combining different data forms.

## Introduction

**Figure 1:**
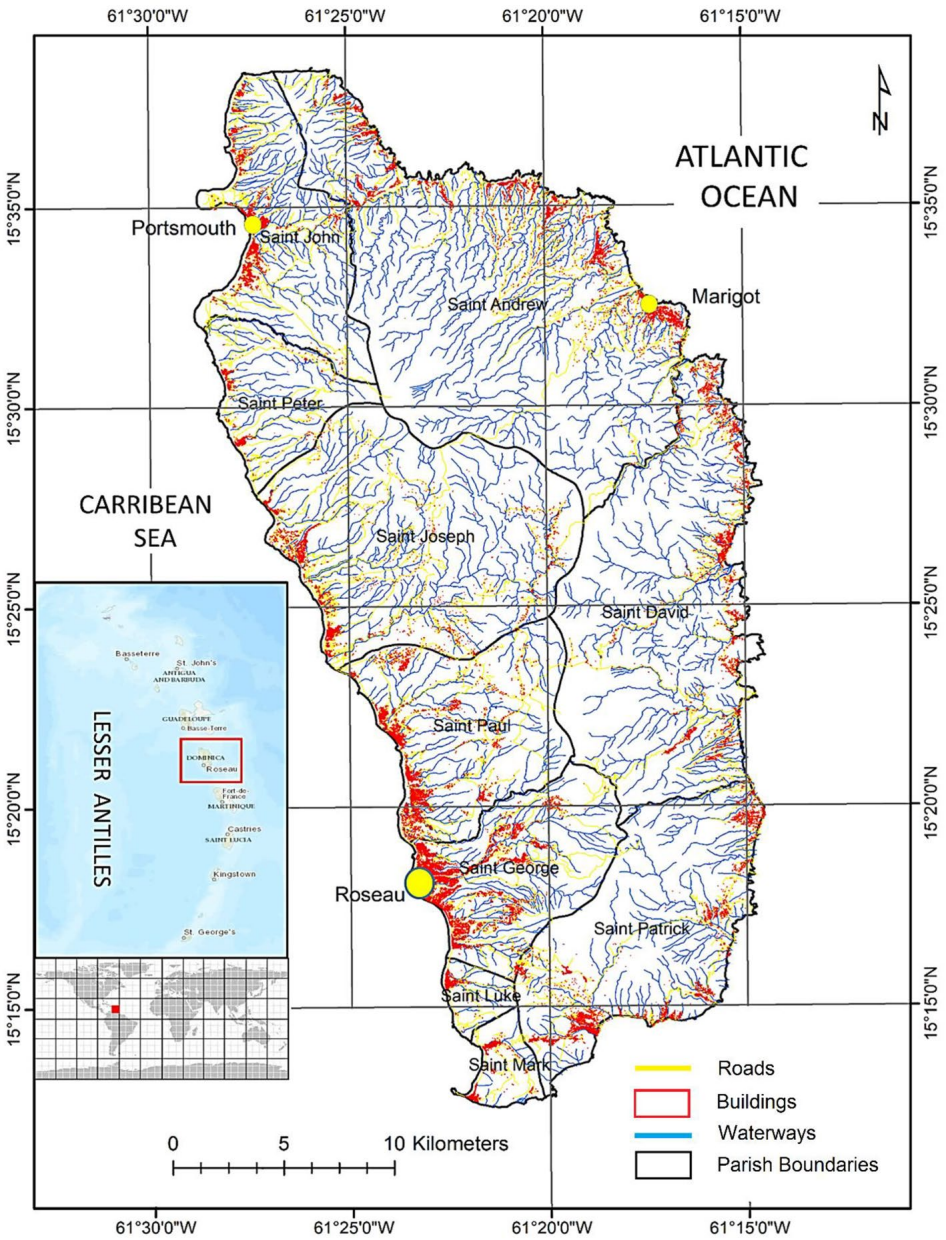
Location of Dominica in the Eastern Caribbean.

The Caribbean experiences severe disasters^1^ with 163 hurricanes and tropical storms reported in the last 2 decades leading to over 5000 deaths^2,3^, affecting 1.2 million people and incurring annual economic losses of US$1.6 billion. Poor and socially vulnerable sections of the communities have been particularly impacted^4^ in small island developing states (SIDS)^5^. Dominica is a small island developing state in the eastern Caribbean (Fig. 1). The island is geologically young and almost entirely volcanic in origin^6^, covering an area of 751 km^2^, with an elevation ranging from 0 to 1447 m and a shoreline of 148 km. The island has a rugged mountainous topography covering 90% of the island with deeply incised valleys and steep slopes^7^. It experiences a warm, humid, tropical climate throughout the year. The average temperature of the island is 29 °C, and the yearly average rainfall ranges from 1900 mm near the coasts to about 7500 mm in the inland mountainous areas. Household-level economic conditions are noticeably uneven; an assessment made during 2008–2009 suggested that 29% of the island’s population was living below the poverty line, which rises to 49% for the Indigenous peoples^8^. A substantial size of the population with low income is living in informal settlements and is vulnerable to multiple natural hazards^9^. Dominica is an example of the intersection of compound multiple natural hazards, high exposure levels, and physical and social vulnerabilities in SIDS exacerbating the impacts.

**Figure 2:**
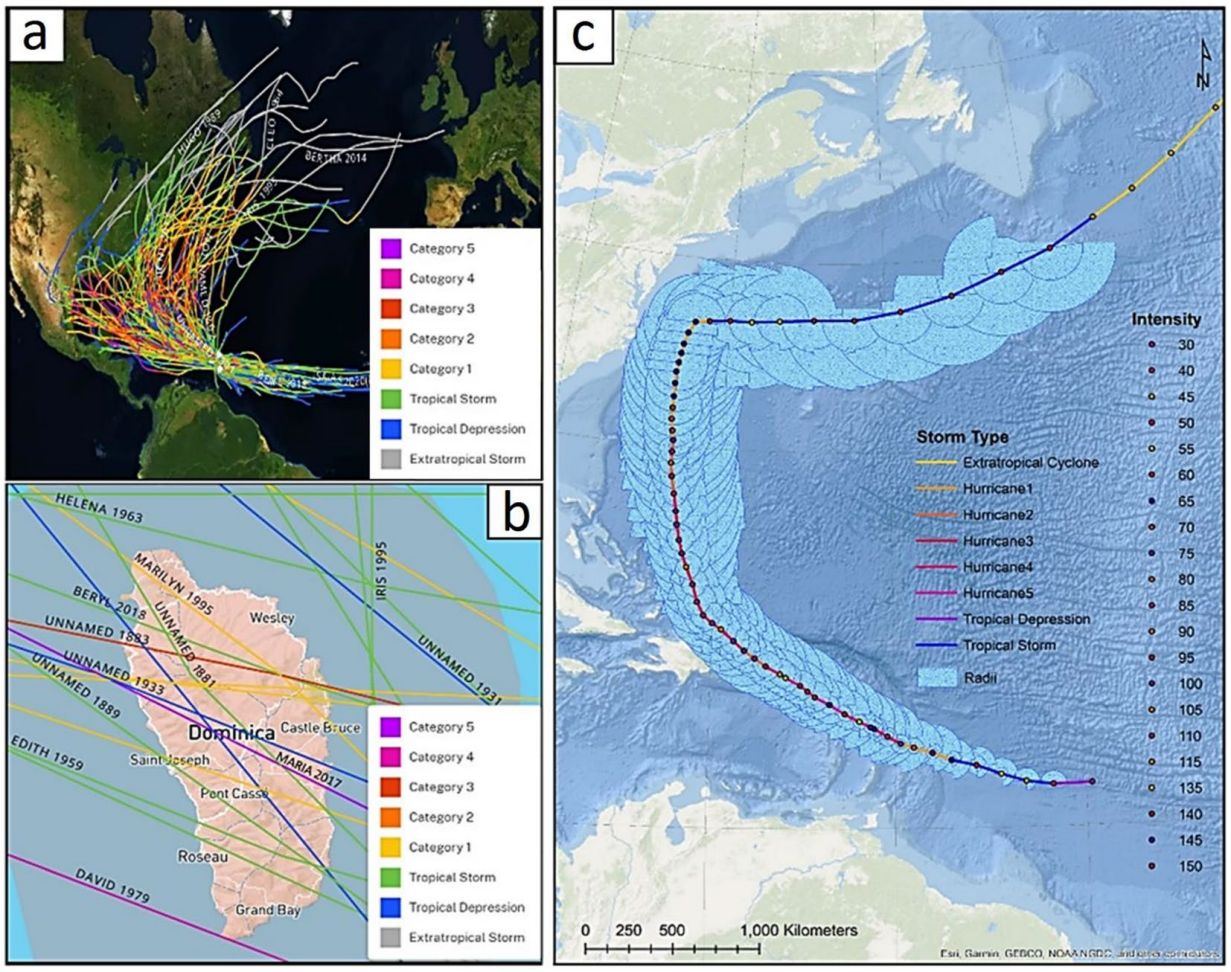
(**a**) Hurricanes within 111 kms of Dominica for the period from 1851 to 2020; (**b**) intensity of the past hurricanes with track over Dominica; (**c**) track, intensity and wind radii of the Hurricane Maria 2017.

**Figure 3:**
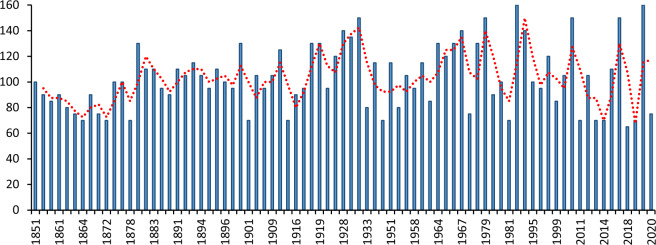
Intensity (kt) of hurricanes within 111 kms of Dominica from 1851 to 2020. The data has been obtained from the National Oceanic and Atmospheric Administration (NOAA) hurricane database.

Dominica has experienced fatalities, damage to infrastructure and agriculture, and livelihood losses due to recurring disasters^10–13^. The island is exposed to a range of natural hazards, and hurricanes have been devastating with lasting impacts owing to their island-wide impact. Hurricanes are generally accompanied by widespread landslides and flooding^14–16^. Dominica has been affected by several hurricanes in the recent past (see Figs. 2 and 3, Table S1). For example, Hurricane David which hit the island on 29 August 1979, caused 42 deaths and 2000 injuries, leaving 78% of the population homeless with huge losses to buildings, roads, agriculture and forests^14,16^. Hurricane Dean (2007), accompanied by severe rainfall^15^, damaged almost all the sectors of the economy and caused estimated losses of US$36.5 million to infrastructure^17^. Tropical Storm Erika which hit in 2015, affected about 23% of the country’s population directly^18^, killing up to 20 people and resulting in economic losses of US$482 million^8^. Even though Erika was a tropical storm with a sustained wind speed of 80 km/h, the associated heavy rainfall of 380 mm in seven hours triggered flash floods and landslides across the island^1,19^. Transportation infrastructure was worst hit in this storm, contributing 60% of the total damage, followed by housing (11%) and agriculture (10%)^8,20^. While the population was still struggling to recover, Hurricane Maria, a category 5 hurricane, made landfall on 18 September 2017, leaving 31 dead and 34–37 missing. The hurricane affected 80% of the population directly and caused estimated damage of about US$1.31 billion^16,21,22^. Maria triggered about 9,960 landslides and caused all the rivers to flood, compounding the impact over the entire island^23^.

Although several studies have been carried out in Dominica^9,23,24^, the lack of information on the nature and level of hazard risks are major obstacles to reducing disaster risks in Dominica^7^. Hurricane-centred multi-hazard risk assessment would serve as a foundation for disaster risk reduction policies and practice^25,26^ and would help in the understanding of spatial patterns of hazards, vulnerabilities, and elements at high risk^27,28^.

Multi-hazard risk assessment is a complex process and considers many criteria^29^. Risk assessment has been approached in different studies by employing independent or coupled hazards and vulnerability factors; however, one common general risk assessment model defines risk as a function of the hazard, exposure to the hazard, and degrees of vulnerabilities^30,31^. The relationship between the components of risk is usually depicted spatially through overlay analysis in GIS by adopting either a qualitative, quantitative, or mixed approach^32^ at scales varying from a community level^33,34^ to regional^35^ and global levels^36^. The assessments do not merely depend on the spatial merger of the variables but consider the interactions between them and their dynamics over a period of time^37,38^. The quantification methods of interactions between the hazards can be broadly classified into stochastic, empirical, and mechanistic types^39–41^. Multi-hazard risk assessments consider either different types of hazards (which do not necessarily occur in chains)^42–44^ or interdependent components of a single hazard^41^. Moreover, the choice of elements for representing the exposures and vulnerabilities varies in different investigations. People’s perception of risk has been an important facet of risk assessment studies. Risk perception research usually focuses on statistical analyses of questionnaire surveys. However, according to Kelman et al.^45^, only quantitative approaches are not able to address the breadth of issues surrounding vulnerability. In reviewing social methodologies for the Caribbean, Mercer et al.^46^ picked out the strengths of participatory approaches, including learning from local knowledge, emphasis on visual techniques and focus on community strengths rather than dwelling on weaknesses. Participatory rural appraisal (PRA) has allowed the lived experiences of local communities to be captured where the local participants decide what goes onto a hazard map and not the researchers^47,48^. PRA methods can, therefore, better capture how communities interpret their social world and social vulnerabilities.

The present study demonstrates the importance of the identification and treatment of risk at different geographic scales in a single framework. It also emphasizes the inclusion of specialized hazard simulation models and community perception in multi-hazard risk assessment studies, which enhances the reliability and operational applicability of the results. In order to understand the likelihood of future disaster impacts under the existing geophysical and demographic conditions, the present study aims to perform a comprehensive hurricane risk assessment in a multi-hazard context for Dominica. The assessment includes developing susceptibility scenarios for the selected hazards, projecting the composite of the hazards, evaluating the underlying demographic and social factors that make the population vulnerable to the impacts of the hazards, and integrating all the factors for projecting the overall risk. The investigation also involves applying dynamic models for simulating hurricane storm surge, landslide runout, and flooding scenarios at specific sites in the study area to answer the questions including: (1) How is storm surge of a particular height likely to affect coastal areas? (2) What would be the landslide runout scenarios for different volumes of debris and under variable friction parameters along a particular slope? (3) What inundation scenarios will emerge for given flood discharge magnitudes at selected river reaches? (4) What do local experts think are the key vulnerabilities to disaster risks? (5) How does the local community perceive accumulated risks and the measures needed to mitigate them?

## Data and methodology

### Study design

Evaluating multiple hazards and assessing their cumulative effects over a region are important dimensions of disaster risk assessment^37,39,40,49,50^ Although several established deterministic and probabilistic approaches exist for assessing the risk from individual hazards, functional frameworks for examining multiple hazards in totality are rarer^38,39,51,52^. Applying multi-hazard risk assessment is challenging due to diverse data requirements and parameterization^39,53,54^. The issues mainly arise from inherent differences in the genesis and spatial scales of the natural hazards, variability in the vulnerabilities that systems exhibit towards each hazard, and temporal changes in the associated elements. The procedural intricacies are also associated with subjectivity concerning the datasets and difficulties in merging variables to simulate composite scenarios of risk from multiple natural hazards^55^.

A comprehensive risk assessment usually involves a spatial characterization of the factors related to the selected hazards (e.g. intensity, frequency, and location) and their interaction with the communities’ vulnerabilities, i.e. the physical and social conditions of people at different scales varying from individual to household and municipal to regional level^30,31,42,55–59^.

**Figure 4:**
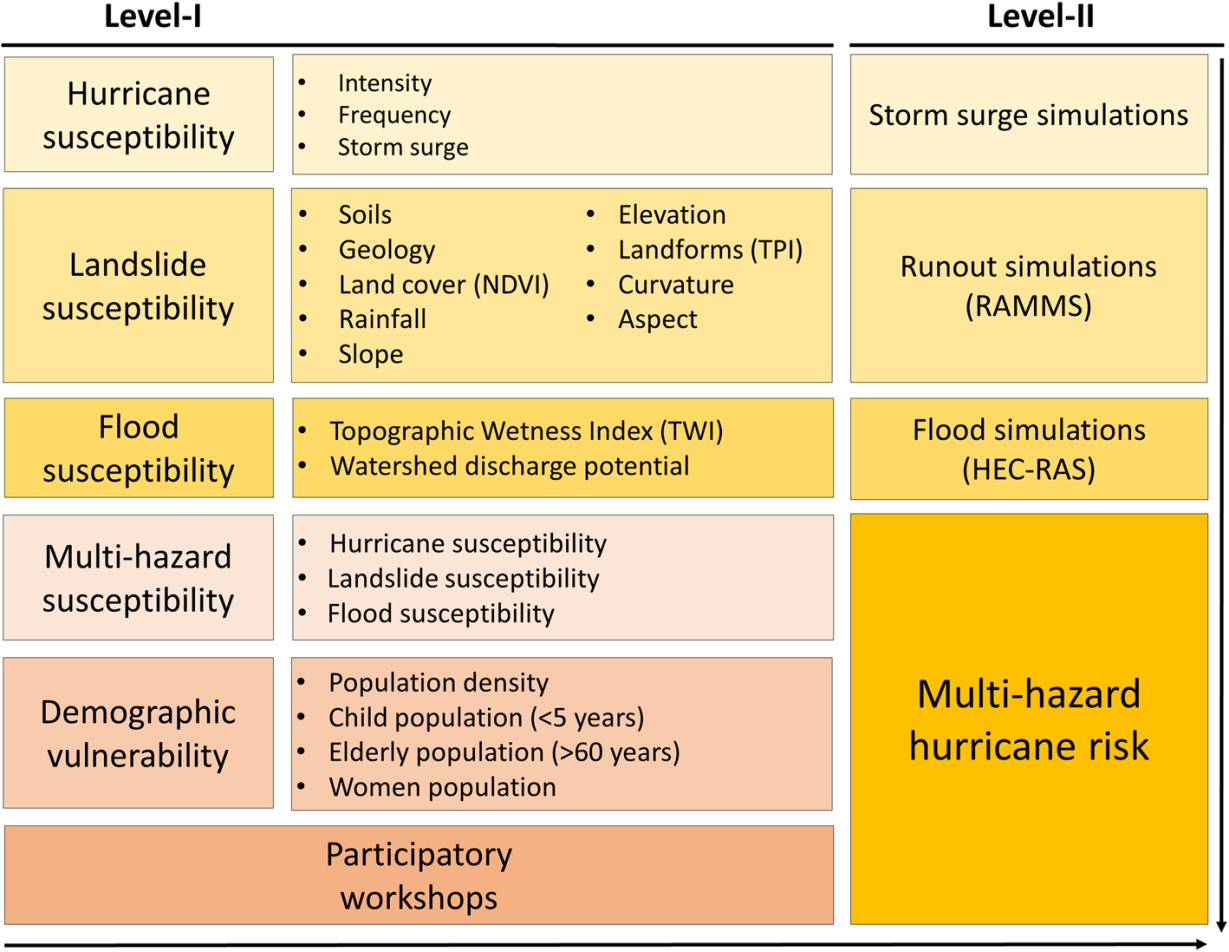
Methodological framework adopted for the present study.

Here, we adopt a modified expression of disaster risk (Eq. 1) for the multi-hazard hurricane risk assessment in Dominica.

1 $$Hr \, = \, Hs \, \times \, Ls \, \times \, Fs \, \times \, Vp,$$

where *Hr* is the hurricane risk, *Hs* hurricane susceptibility, *Ls* landslide susceptibility, *Fs* flood susceptibility, and *Vp* vulnerable population (see Fig. 4 for full details on the adopted methodology).

The assessment was carried out at two spatial scales: (1) regional scale and (2) local scale, which we name Level-I and Level-II, respectively. The Level-I analysis developed broad susceptibility scenarios of hurricane, landslide, and flood hazards using a range of physical parameters derived from multiple moderate-resolution datasets. The multi-hazard status of each location is derived from the reclassified values of the selected data layers with consistent projection (WGS 1984/UTM Zone 20N) in GIS. The composite of the susceptibilities was then merged with the final vulnerability map to project the hurricane risk for the entire island of Dominica. The Level-II analysis has been site-specific, modelling diverse scenarios of the selected hazards.

### Multi-hazard susceptibility analysis

#### Hurricanes

**Figure 5:**
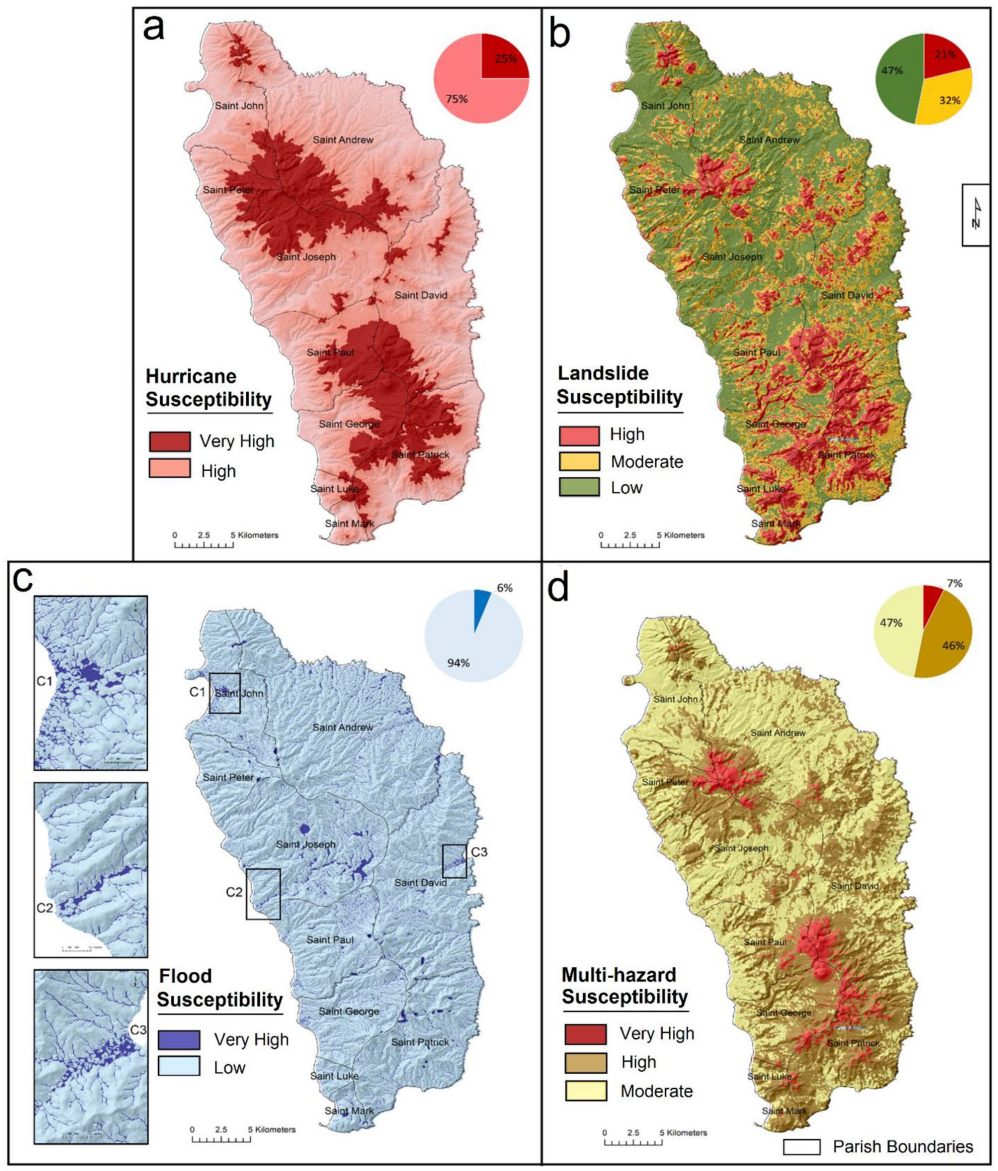
Multiple hazard susceptibility of Dominica: (**a**) hurricanes; (**b**) landslides; (**c**) floods; (**d**) composite hazard scenario of all the selected hazards. Pie graphs show area under each hazard category.

Wind speed, rainfall, and storm surge are three essential attributes of hurricanes that describe their damage potential^60–63^. During hurricanes, high-elevation mountainous areas usually experience extreme rainfall due to orographic controls^15,64^, whereas low-elevation locations along the coasts experience impacts from storm surges^60,64^. Orographic precipitation is considered a key issue for Dominica and exhibits two general patterns over the island: (1) convection over the windward east coast, which is responsible for most of the precipitation; (2) precipitation accumulation increases with an increase in elevation^62^. However, some inconsistencies from the general trends exist, such as precipitation accumulation from Erika did not show significant orographic control: low-elevation coastal gauges and high-elevation mountain gauges received similar amounts of rainfall^65^. While the patterns of rainfall and storm surge associated with hurricanes can be determined with a high degree of confidence, the probabilistic assessment of the hurricane intensity is difficult for a small island such as Dominica. In this analysis, elevation is taken as the primary criterion to establish the spatial variability in rainfall and storm surge (Fig. 5a). In summary: (1) Hurricane intensity and frequency are assumed constant for the whole island; (2) rainfall increases with an increase in elevation during an event; (3) storm surge impacts are restricted to coastal areas below 4 m elevation.

#### Landslides

Multiple factors, including soil, geology, vegetation (characterized by the Normalized Difference Vegetation Index, NDVI), rainfall patterns, slope steepness, elevation, topographic position index (TPI), curvature and aspect (slope orientation) were considered for the landslide susceptibility analysis^66,67^. The contributions of each of these factors (Fig. S1) to landslide susceptibility were determined using the Frequency Ratio (FR) model (see Table S2 in the supplementary material). FR statistical measure is based on the observed relationships between the distribution of landslides and the factors influencing landslide occurrence^68^. In other words, FR determines how the selected environmental conditions favour or restrict landslides incidence^69^. The frequency (FR) ratio of each landslide factor class was calculated using Eq. (2):

2 $$FR=\frac{Npix({S}_{i})/\sum Npix\left({S}_{i}\right)}{Npix({N}_{i})/\sum Npix\left({N}_{i}\right)},$$

where *Npix (S*_*i*_*)* is the number of observed pixels with landslides in factor class *i*, *Ʃ Npix (S*_*i*_*)* is the total number of pixels with landslides, *Npix (N*_*i*_*)* is the total number of pixels in factor class *i*, *Ʃ Npix (Ni)* is the total number of pixels in the entire area of interest (AOI). A value greater than 1 implies a higher correlation and a value lower than 1 suggests a lower correlation. The landslide susceptibility of an area is then derived from the summation of the frequency ratios (Eq. 3) of all the selected landslide-causing factors^70^.

3 $$LSI={FR}_{1}+{FR}_{2}+{FR}_{3}+\dots {+FR}_{n}.$$

In order to apply the FR model in landslide susceptibility analysis, the inventory of previous landslides experienced in Dominica is needed as an input. In total, we used 2134 landslide events. 1829 events mapped for the period of 1988 to 2014 were obtained from the Charim Project (www.charim.net), and 305 events were digitized from multi-temporal Google Earth scenes (2017 to 2020). About 75% of the landslide events were used for training purposes and 25% for testing the accuracy of the generated landslide susceptibility map; the accuracy of the derived landslide susceptibility map was assessed (Figs. 5b and S2) using the landslide density index (LDI) which calculates the ratio between testing samples and the degree of susceptibility^68–70^.

The level-II analysis for landslide hazard was performed at specific locations using the Rapid Mass Movement Simulation (RAMMS) model. RAMMS has been applied to varied sizes of debris flows ranging from less than 1000 m^3^ to a few million m^3^^71^. The model includes three different components, i.e. avalanche, debris flow, and rock fall. The debris flow module is used to calculate the motion of movement from initiation to runout in three-dimensional terrains; RAMMS uses depth-averaged equations and predicts the slope-parallel velocities and flow heights^72^. The model employs the Voellmy friction law that splits the frictional resistance into the dry-Coulomb type friction *μ* (Mu) and the viscous-turbulent friction *ξ* (Xi)^71^. The frictional resistance S (Pa) is then calculated as Eq. (4):

4 $$S=\mu N+\frac{\rho g{u}^{2}}{\xi }withN=\rho hgcos\left(\phi \right),$$

where, ρ is the density, g the gravitational acceleration, φ the slope angle, h the flow height, and u the vector u = (u_x_, u_y_)^T^, consisting of the flow velocity in the x- and y-directions. The normal stress on the running surface, ρhgcos (φ), can be summarized in a single parameter N^72^.

#### Floods

The Level-I flood susceptibility assessment made in the present study (Fig. 5c and S3) is based on the topographic wetness index (TWI) derived using a 12.5 m digital elevation model (DEM). Developed by Beven and Kirkby^73^, the TWI is an efficient algorithm used for multiple purposes ranging from soil moisture estimation^74^ to delineating areas prone to flooding at the catchment scale^75,76^. The index is used as an indicator to assess the control of topography on flood water direction and accumulation and is calculated as: I = ln(a/tanβ), where ‘I’ is the index value, ‘a’ is the upstream contributing area draining through a certain point, and ‘tanβ’ is the local slope^77^.

Determination of Level-II flood susceptibility involved the steady flow analysis of a selected stretch of Roseau River using the Hydrologic Engineering Center (HEC) River Analysis System (RAS). HEC-RAS is a combined assessment with four hydraulic analysis modules: (1) steady water profile calculations; (2) unsteady flow simulation; (3) movable boundary sediment transport estimation; (4) temperature and water quality constituent modelling^78^. The computation of water surface profiles assumes a steady flow scenario. The method is based on an iterative solution of the energy equation (Eq. 5):

5 $$H=Z+Y+\frac{{\alpha V}^{2}}{2g}.$$

The equation states that the total energy (H) at any given point along the river is the sum of potential energy (Z + Y) and kinetic energy ($\alpha$ V^2^/2 g)^78^. HEC-RAS has tremendous capability to simulate flood hazard conditions and has been used widely for flood hazard modelling in a variety of settings^79,80^. The application of the HEC-RAS is particularly useful for designing hydraulic structures along rivers and for the management of floodplains.

#### Multi-hazard susceptibility

The composite of multi-hazard susceptibility of the study area was generated by superimposing the derived susceptibilities of the selected hazards, i.e. hurricanes, landslides, and floods (see Fig. 5d) through the overlay function in ArcMap 10.2 using weights determined through the Analytic Hierarchy Process (AHP)^52,54,55,81^. Understanding the weight or percentage of influence from each hazard is important because the selected hazards contribute variably to the overall susceptibility or risk level. AHP is extensively used to help in making such decisions (Table S3) about the priorities^55,82^. It involves three basic steps: (1) Identify the problem and determine what type of information is to be obtained; (2) construct a pairwise comparison matrix; (3) use the priorities obtained from the comparisons to weigh the selected factors^82^. To be sure about the rationality of decisions made during the process of pairwise comparisons of the selected criteria, AHP has to go through a consistency check which is done by calculating the consistency ratio (CR). The CR is expressed as Eq. (6):

6 $$CR=\frac{CI}{RI}.100\%,$$

where, CI is the consistency index (CI), calculated using Eq. (7):

7 $$CI=\frac{\left({\lambda }_{max}-n\right)}{n-1},$$

where λ_max_ is the highest eigenvalue of the matrix and *n* represents the size of the matrix; RI is the random index representing the consistency of a randomly generated pairwise comparison matrix. The comparisons can be considered suitably consistent if the CR is ≤ 10%^82^.

In this analysis we applied AHP to determine the relative importance of the selected hazards and quantify the multi-hazard susceptibility^81^. Since the hurricane hazard is widespread with long-lasting impacts and acts as a trigger for the other hazards, it is given a ‘strong importance’, followed by landslides with a ‘moderate importance’ over floods. As a result, the relative dominance of hurricanes, landslides, and floods on the overall susceptibility scenario is 30.3%, 10.2%, and 6.4%, respectively. The statistical details of the AHP are presented in Table S4.

### Vulnerability assessment

Demographic factors are among the primary considerations for risk simulations. Here we use four sensitive demographic variables as the indicators of vulnerability^83,84^: (1) population density; (2) child population under 5 years; (3) elderly population above 60 years; (4) female population (gender). The selected demographic factors have revealed a positive relationship with the fatalities during past extreme events almost around the world^83,84^. For instance, the Dominica Census (2011) shows a strong correlation between gender and health inequalities of non-transmissible diseases (e.g. hypertension and diabetes) and income inequality. While age above 65 years shows a strong correlation with disability. The data on demographic attributes are for the year 2020, obtained from the OCHA database^85^. These four are the only variables where the data are disaggregated at the parish level. Although, a population density of 96 persons per square kilometer in Dominica may seem low, the distribution of population is highly uneven. Most of the population (61%) is concentrated in coastal areas, while the interior mountainous areas of the island remain largely uninhabited. In general, the areas around Roseau in the southwest and Portsmouth in the northwest have the highest population density (Fig. S4). The distribution of population sets the spatial patterns of other factors, such as child population, elderly population, and female population, approximately the same as observed for population density (Fig. S4). Among the vulnerability parameters, the AHP priority from highest to lowest has been in the order of child population, population density, elderly population, and women population with a contribution of 23.8%, 12.6%, 9.1%, and 7.5%, respectively. The overall consistency ratio (CR) of the comparisons was 7.0%. The AHP-derived relative contribution of each factor was used as input for weighted overlay analysis in GIS for mapping the spatial risk scenario of the entire Dominica.

### In-depth risk perception of the local experts and population

Qualitative interviewing with key experts allows an understanding of past responses and risk perception in relation to development and sustainability. Our research focused on two key questions: (1) What are the key vulnerabilities of people to hurricanes and associated hazards? (2) How does an infectious disease epidemic affect these? To address these, both semi-structured interviews of local key informants and participatory rural appraisal (PRA) focus group discussions were employed to understand local risk perceptions, vulnerabilities and organisational response to disasters (see Table S5, Appendix 1, 2 & 3). Before conducting the research, all ethical approvals were obtained from the *UCL Research Ethics Committee* in accordance with the relevant institutional (University College London) guidelines and regulations. Accepted ethical standards included informed consent, benefit not harm, and confidentiality. 17 thirty-minute semi-structured interviews were conducted with key informants in Dominica between June and July 2021. An interview guide was first prepared, and a pilot conducted. The key informants were local experts selected with the aim of achieving a breadth of opinion. They were drawn from the education sector, agriculture and fisheries, health, business, social services and welfare, government finance and lifeline offices and local government and included experts on gender, the blue and green economy, development, the environment and disaster management. The interviews were conducted by local partner organisations after training from UCL researchers.

Four half-day participatory workshops were held in June and July 2021 using facilitation teams from the Dominica Red Cross and IsraAID, trained by UCL researchers. One workshop was in the capital Roseau on the south-west coast, two in the second town Portsmouth on the north-west coast and one in the village of Layou on the west coast. Roseau is the largest city in Dominica, with a population of 14,725. It is the most important trading port for foreign trade with a large service sector. Portsmouth is the second largest town with 2977 inhabitants. It has its own seaport. Layou is a small fishing village on the Layou River. Workshop participants were recruited by the Red Cross and IsraAID from their networks. There were about 30 participants in total, covering local adults and representatives from the local government, professionals, business people, fishermen, and farmers. In Layou, where the workshop was sufficiently large, the participants were divided into women’s and men’s groups. The workshops focused on past hazard events, their priorities and solutions and aspirations for development. The facilitators explained the workshop activities, participatory rural appraisal, and ethical issues, and took informed oral consent from the participants.

PRA approaches help in obtaining information on local knowledge, people’s risk perceptions, contextual factors, and local responses in mitigating hazard risks^48^. Workshop participants prepared two maps: (1) Hazard perceptions maps, including resources and issues of disaster vulnerability and exposure; (2) Dream maps, where participants draw maps to illustrate their aspirations for building resilient communities. Two groups generalised these maps for the whole of Dominica, three groups detailed their local areas. Facilitators asked the participants to explain their maps and field notes were taken.

The outcomes of the risk perception of the local experts and population were used to inform the weightings of the vulnerability indicators.

### Informed consent

Informed consent was obtained from all interview and workshop participants.

## Results

### Hurricane susceptibility (level-1)

Based on the criteria described in Section “Hurricanes”, two broad hurricane hazard zones were identified in Dominica (Fig. 5a): (1) ‘very high’ hazard zone spread over elevated mountainous areas (>500 m), constituting 25% of the island and (2) ‘high hazard’ zone that constitutes remaining 75%. Notably, no sharp boundary exists between the identified hurricane hazard zones and the demarcation should be considered as an approximation.

#### Storm surge simulation (level-II)

**Figure 6:**
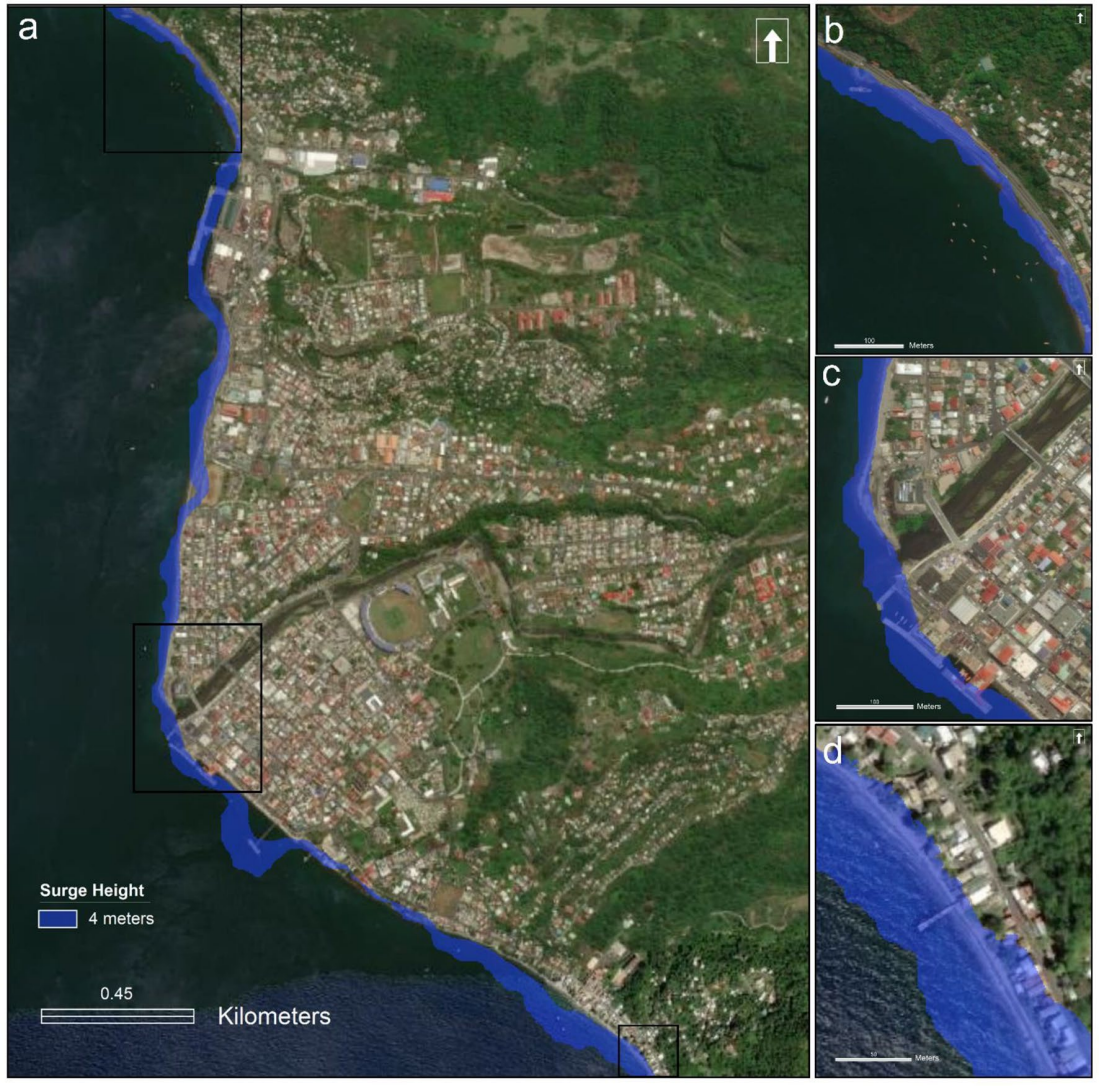
Storm surge scenario likely to emerge under 4 m water column, derived using Digital Terrain Model (1 m) and Google Earth data. The figure shows the areas that may submerge under the given surge conditions. Inset (**b**), (**c**), and (**d**) show the zoom-in view of the locations within black colour rectangles on the part ‘(**a**)’ of the figure.

The level-II analysis for the hurricane hazard is restricted to storm surge simulation only. A storm surge height of 3.7 m was observed during Hurricane Maria in 2017^86^. Considering the experienced surge height, we simulate a scenario which may evolve under the 4 m surge height. The coupled use of the high-resolution digital terrain model and optical satellite imagery has been used to identify the areas expected to be affected during storm surge of the given size (Fig. 6). Along the coast of Roseau, a 4 m surge may inundate the main road at several locations and cause disruption in transportation along this main corridor around the island. Elevations along the main coastal highway were confirmed by a ground-truthing survey. Field validation indicated the main coastal road is about 3.5 m above the high tide mark and protected by a 1 m sea wall in the north of Roseau; 2 m above the high tide and protected by a 1.5 m sea wall in central Roseau; 4 m above high tide and unprotected south of Roseau. We also map 8 m high scenario of surge to identify the areas likely to get affected by a surge height double the size experienced in 2017 during Hurricane Maria (Fig. S5).

### Landslide susceptibility (level-I)

The FR-based landslide susceptibility analysis categorized the area of interest manually into three custom class ranges i.e., low, moderate and high with an area of 47%, 32% and 21% respectively, where the probability of landslide occurrence is maximum in the identified high susceptibility class (Fig. 5b). The accuracy of landslide susceptibility predicted through the FR model has been fairly good (Fig. S2). Landslide density index (LDI) reveals that the density of landslide testing samples has been highest (2.38) in the identified ‘high susceptibility’ class, and least (0.34) in the ‘low susceptibility’ class. The results reveal that the high-elevation areas of Saint George Parish, Saint Patrick Parish, Saint David Parish from the south, and Saint Andrew from the north are highly prone to landslides. In addition, steep slopes along the river channels within the island and along the coast, particularly in the south, are the areas exhibiting high landslide susceptibility.

#### Landslide runout simulation (level-II)

**Figure 7:**
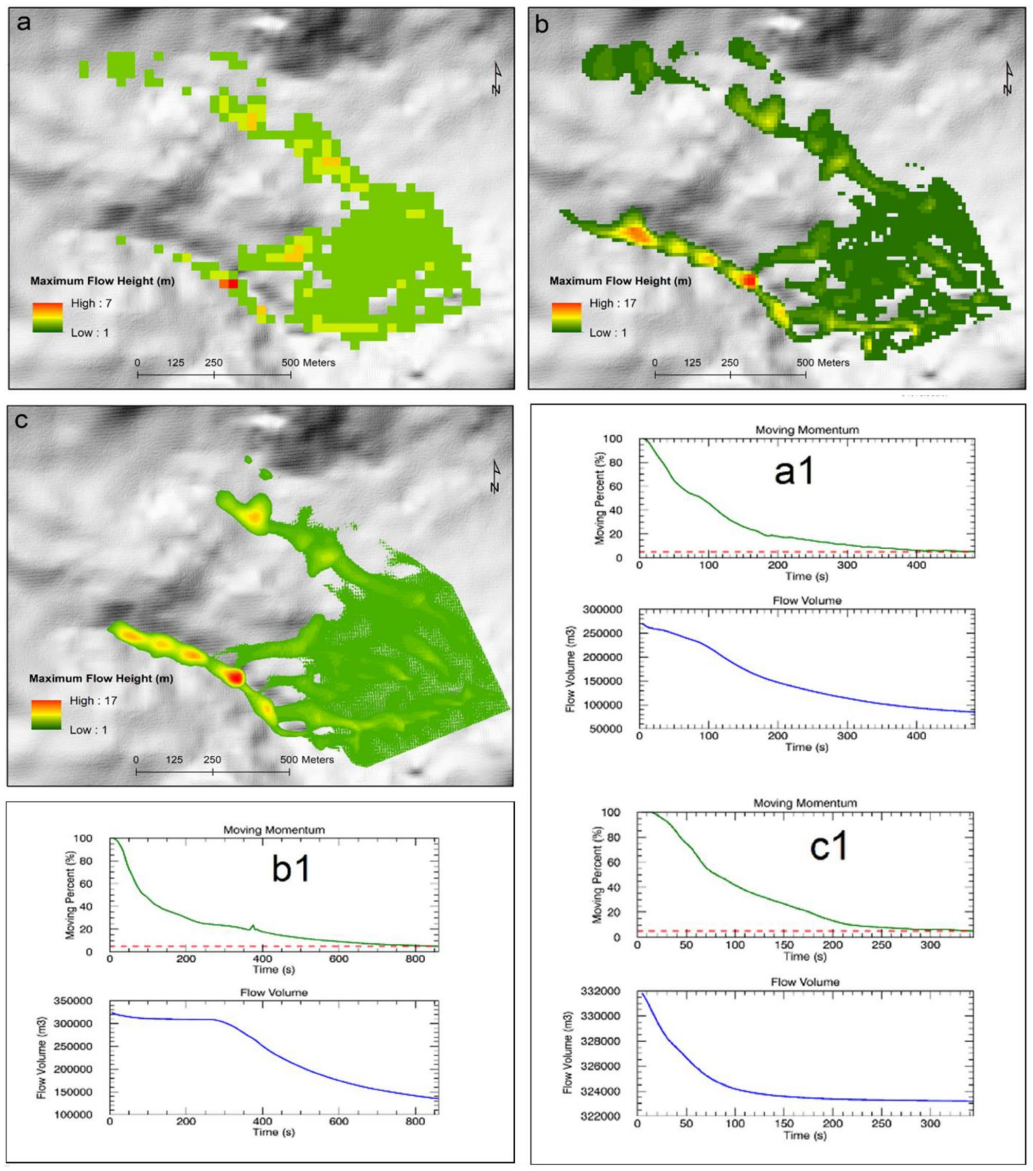
RAMMS simulation results obtained under variable spatial resolution of digital topography products. This figure shows the variability in flow height, flow volume, and moving momentum of the landslides under different spatial resolution digital elevation models: (**a**) Shuttle Radar Topography Mission (SRTM) 30 m, (**b**) Advanced Land Observing Satellite (ALOS) Phased Array type l-band Synthetic Aperture Radar (PALSAR) 12.5 m, (**a**) NEXTMap One Digital Terrain Model (1 m); (**a1**), (**b1**), and (**c1**) show the flow momentum and flow volume corresponding to (**a**), (**b**), and (**c**) respectively.

**Figure 8:**
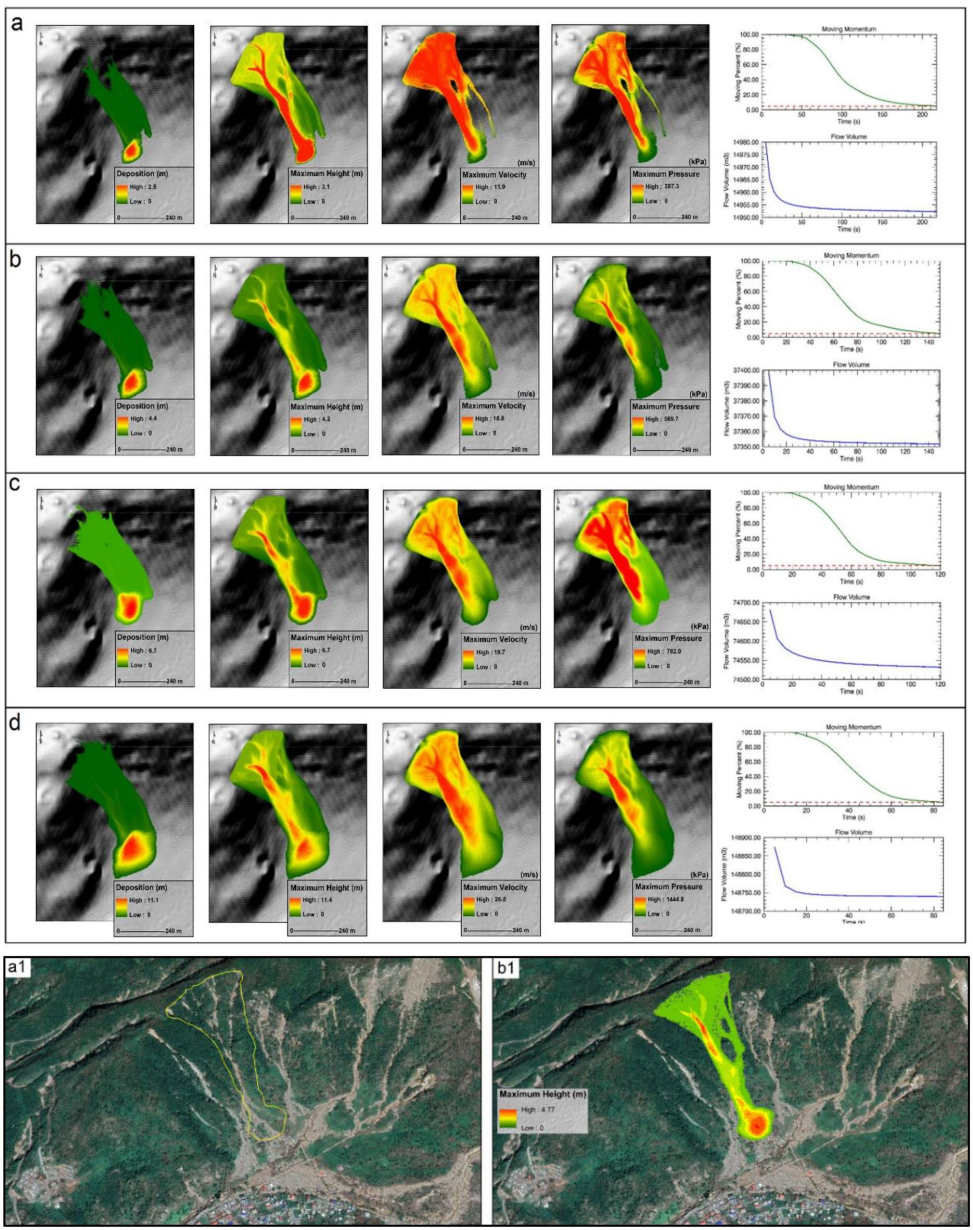
Landslide runout simulations. Spatial patterns of deposition, maximum height, maximum velocity, maximum pressure, flow volume and moving momentum of debris under different friction parameters (*μ* and* ξ*) and release depth volumes ((**a**) 0.5 m, (**b**) 1.0 m, (**c**) 3.0 m). (**a1**) location of the selected slope and (**b1**) simulation results of 0.5 m release depth volume overlain on Google Earth image.

High-resolution landslide simulations have tremendous potential in identifying unstable slopes, initiating mitigation measures and minimizing landslide impacts in the mountainous areas of Dominica. The primary inputs considered for the numerical calculation in RAMMS include, (1) digital elevation model (DEM), (2) release area, and, (3) model friction parameters^87^. In this analysis, we used three different terrain data resolutions in order to assess the impact of data quality on the simulation results. The DEM resolution has a substantial impact on the quality of results and computation time^87^. The DEMs employed were—Shuttle Radar Topography Mission (SRTM) 30 m, Advanced Land Observing Satellite (ALOS) Phased Array type l-band Synthetic Aperture Radar (PALSAR) 12.5 m and NEXTMap One Digital Terrain Model 1 m. With constant friction parameters (*μ* and* ξ*) and debris flow release depth, the estimates of release volume, velocity, flow height and pressure from the three different spatial resolutions were analysed and quantified (see Fig. 7 and Table 1). The debris release volume (m^3^) was seen to be highest in the 12.5 m resolution DEM and lowest in the 30 m resolution DEM. Other parameters including velocity (m/s) and flow height (m) were consistently higher for the fine resolution (1 m) terrain data than the coarser resolution products (Table 1). Overall, the high-resolution data produced better and spatially coherent results than the coarser topography data (Fig. 7). Moreover, the computation time differed from a few minutes to tens of minutes for the coarse and fine spatial resolution data respectively. We also simulated the runout conditions under variable friction parameters and release depths. The spatial patterns of deposition, maximum height, maximum velocity, maximum pressure, flow volume and moving momentum of debris under different friction parameters (*μ* and* ξ*) and release depth volumes are shown in Fig. 8 and Table 2. The simulation results can be useful for designing and developing structures and safety measures along the mountain slopes prone to landslides.

**Table 1:** Effects of the digital elevation model (DEM) resolution on release volume, velocity, flow height and pressure of the debris flows.

Simulation resolution (m)	Release volume (m^3^)	Velocity (m/s)	Flow height (m)	Pressure (kPa)
1.00	334,439	16.66	17.86	555.26
12.49	345,315	15.64	17.91	489.51
30.48	318,490	11.16	7.69	249.13

**Table 2:** Simulation results under different friction parameters (Mu, Xi) and release depth volumes.

Release depth volume	Mu ()	Xi (m/s^2^)	Calculated release volume (m^3^)	Overall MAX velocity (m/s)	Overall MAX flow height (m)	Overall MAX pressure (kPa)
0.2 m	0.10	200	15,124.8	11.98	3.10	287.33
0.5 m	0.10	200	37,812.1	16.87	4.76	569.71
1.0 m	0.20	200	75,624.2	19.77	6.31	782.00
3.0 m	0.10	200	151,248.0	26.87	11.80	1444.95

### Flood susceptibility (level-I)

The coast of Dominica mostly comprises small watersheds with not well-defined divides (Fig. S3a); because of their small size, these watersheds have less flow generation potential compared with interior mountainous watersheds with greater size^88^. The steep, larger watersheds have high water delivery capability during rainfall events, making the downstream areas along their rivers prone to floods (Fig. S3). The localised terrain condition, i.e. low-lying concave geometry, is also an important factor governing the impacts of floods. The application of TWI allowed us to identify the locations that favour accumulation and higher inundation depth during flooding. In Fig. 5, part ‘c’, zones classified as high flood hazard from this analysis, constituting 6% of the study area, are marked with dark blue. TWI also identifies volcano vents and lakes as flood-prone areas because of the sensitivity to terrain morphology; such locations have little anthropogenic activity beyond limited tourism and are less likely to experience any loss of lives and property. However, these locations may pose a serious flood risk to the downstream population in case of damming and subsequent breaching or spillover.

#### Steady river flow analysis (level-II)

**Figure 9:**
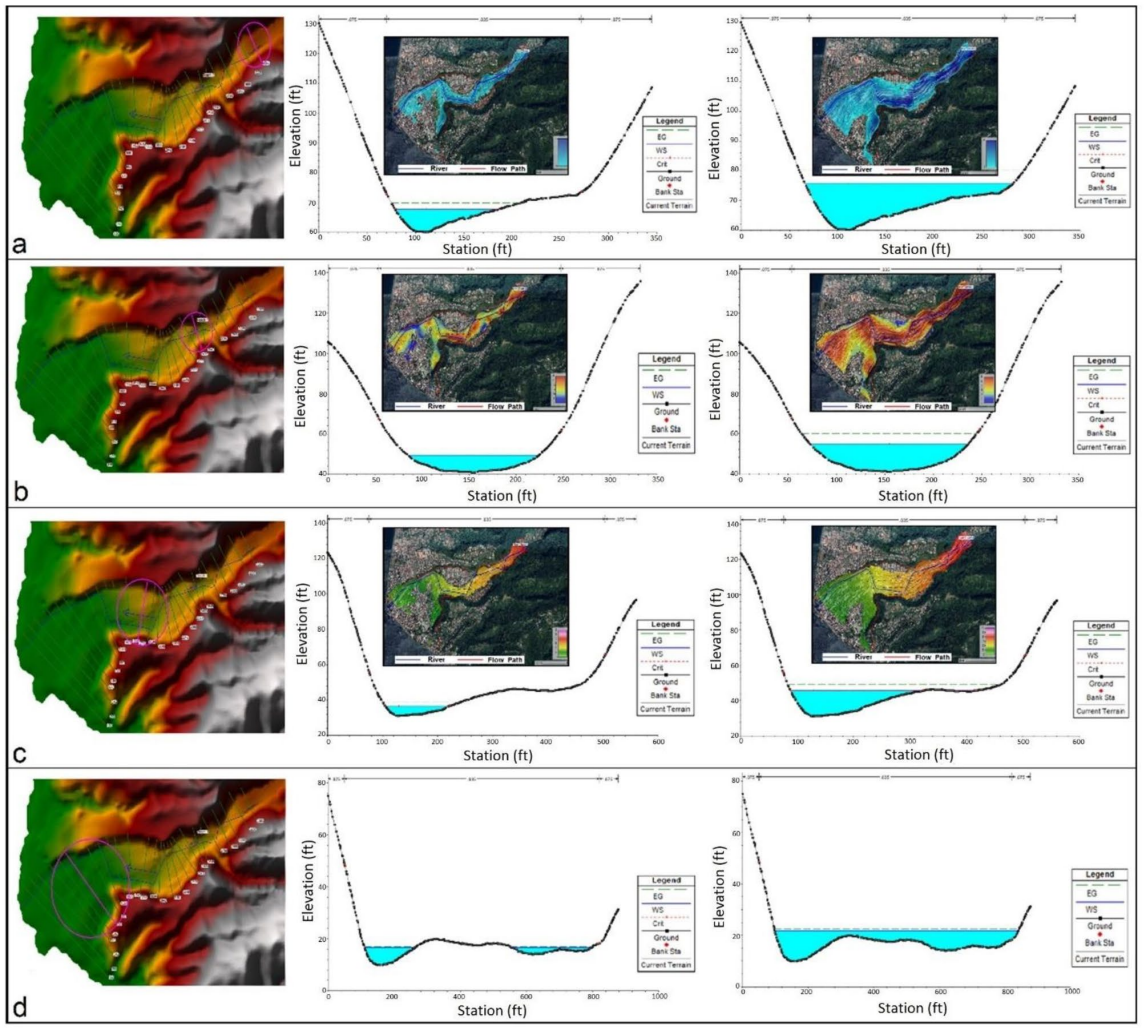
Steady flow simulation of the Roseau River using HEC-RAS. 3D terrain maps on the left show the location of particular cross-sections along the selected river reach; graphs show the water elevation at the highlighted cross-sections for Q 142 m^3^ (middle) and Q 850 m^3^ (right) respectively. Google Earth images depict the spatial patterns of water depth (**a**), velocity (**b**) and water surface elevation (**c**) for the given discharge rates. Arrows show the flow direction.

Since hurricanes are usually accompanied by flooding across Dominica, we performed a steady flow analysis along a selected reach of the Roseau River, employing HEC-RAS. A fundamental input for steady flow analysis is a Digital Terrain Model (DTM) which in this case is a high-resolution 1 m DTM and a moderate resolution topographic data product (ALOS 12.5 m). The geometry of the selected river stretch, i.e. centre-line, bank lines, flow paths, and river cross-sections, were derived using optical satellite images and DEMs. Standard roughness coefficients for mountain streams with rocky beds and large boulders for both banks (0.075) and for the channel (0.035) were used. Assumed discharge rates of Q 142 m^3^/s and Q 850 m^3^/s were used as inputs for the simulation as they cover a range of flood discharge scenarios. The steady flow analysis simulates flow depths, flow velocities, and water surface elevations (Fig. 9) that may arise during floods of the given magnitudes. The simulation results in combination with high-resolution satellite data, allow the identification of each building likely to be affected during the given flood scenarios. The results of the 1 m DTM were noticeably superior to simulation results produced from 12.5 m topography data. Although very high-resolution LiDAR data are a better choice for this type of analysis if available, the results from 1 m and 12.5 m resolutions are of general use for engineered structures along the river segment and land use planning along the banks (Figs. S6 and S7).

### Multi-hazard susceptibility and risk (level-I)

The present analysis identified the locations that may experience the cumulative impacts from the hurricane multi-hazards in Dominica. Around 7% of the island exhibits relatively high susceptibility, 46% moderate susceptibility, and 47% low susceptibility to multiple hazards. In general, multi-hazard susceptibility mapping reveals an irregular spatial pattern: high elevation areas in the south and the low-elevation zones along the rivers and the coast are predominantly susceptible to multiple hazards. The pattern reflects that multi-hazard susceptibility is dominated by the input factors that are common to all the selected hazards; for example, one of the landscape features common to all the hazards is elevation, which has been a determinant factor for shaping the susceptibility to storm surge effects, orographic rainfall and river overflow. Elevation has also been an influencing factor for FR values in landslide susceptibility because the landslides were observed to have occurred mostly in high-elevation areas of Dominica.

**Figure 10:**
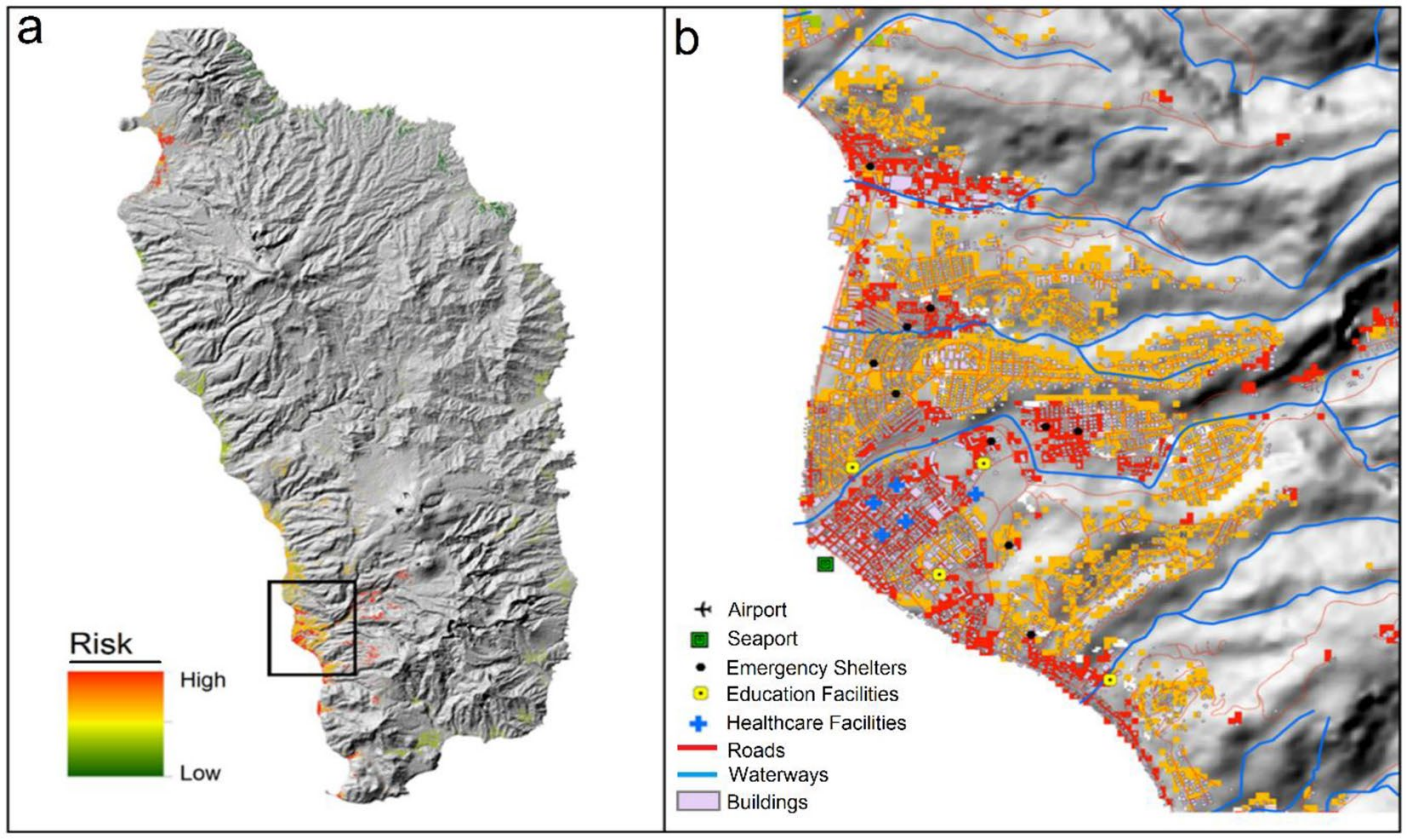
(**a**) Risk to population from the multiple hazards and (**b**) zoom-in view of the area shown in the box on a.

The risk simulation is delimited to populated areas only. On the basis of a composite of multi-hazard susceptibility and demographic factors, the simulation identifies spatial patterns of risk. The projected risk scenario demonstrates the connection of multiple hazards and vulnerabilities and the probabilities of impact on the population and the built environment during future hurricanes. The population exhibits varying levels of risk with differential influences from multiple factors related to the hazard conditions and demographic attributes (Fig. 10). Generally, risk is highest in coastal areas where the population and infrastructure are concentrated. This analysis reveals that the anticipated hurricane events may seriously disrupt the functioning of critical service centres and cause substantial damage to the vital infrastructure in the multi-hazard susceptibility zones. However, impacts from hurricanes are not limited to the population and built environment, as agriculture and natural vegetation may also suffer heavy damage.

### Expert interviews

Inductive coding was employed to identify themes in the data (Tables 3 and S5). Of the experts interviewed, 14 out of 17 identified multiple natural hazards as a key issue. The other three identified urban poverty, crime and housing, and rural land ownership and availability of resources as the key issues. 9 experts identified infectious diseases as a key issue, mostly for COVID-19 but also for Zika, diseases transmitted by the *Aedes aegypti* mosquito and HIV. The interaction of these risks was noted with, for instance, an education sector expert explaining how COVID-19 had not only interrupted education in general but also education for Disaster Risk Reduction (DRR). An expert from the health sector noted how after Tropical Storm Erica and Hurricane Maria, many people lost their livelihoods. Some turned to sex work, and unlike established sex workers, they engaged in riskier sex and were more reluctant to come to the HIV unit. Post-disaster also saw an increase in unhealthy coping mechanisms such as alcohol and drug use. However, health services in Dominica responded to these new situations.

**Table 3:** Interview topics with local experts.

	Main topic	Prompt
1	Main challenges facing Dominica citizens in rural and urban locations	Prompts given on natural hazards and infectious diseases
2	How challenges differ between different groups within Dominican society	Prompts given on men/women, age
3	Kind of support their organisation offers/delivers	Prompts given on implementation, measures of success, effect of Covid-19, data needed
4	Self-support or other non-conventional approaches observed	Prompt for more information if observed
5	Does their organisation engage with these approaches?	Prompt for more information
6	Meaning of resilience	Prompt on relation to their programme
7	Further points on multiple hazards and infectious diseases	Prompt if anything overlooked

In terms of physical vulnerabilities, access to water and food security were the most discussed issues by 6 experts. But also, disaster impacts on infrastructure (roads and the internet) and housing were raised. 14 out of 17 experts across all fields, identified issues around social inclusion, social justice and inequality as key drivers of vulnerability. That this was such a recurring topic is worthy of note. These were in relation to the elderly, youth, women and sexuality. An expert on gender commented on how disasters and the pandemic tended to exacerbate work and financial inequalities between men and women. However, women were more likely to have stronger social networks for support. Homosexuality is illegal in Dominica, and this increases exclusion and heightens vulnerabilities.

Most interviewees understood and discussed the importance of disaster resilience with firm views of what that meant in their sectors. An area of disagreement was to the extent that self and mutual support, as opposed to support from organizations, were needed. Amongst organised religions, increased volunteerism and community support post-disaster were noted. However, one expert from the business sector strongly argued that very often, communities cannot assist themselves, and self-support initiatives are non-evident.

Some specific issues related to emergency planning were raised, such as emergency supplies being centralised when access to outer districts is compromised; the need to update emergency plans in light of the COVID-19 pandemic; and lack of human resources, particularly outside of Roseau and Portsmouth.

#### Participatory mapping

All workshop participant groups identified coastal surges and river flooding (associated with hurricanes) and coastal rockfalls as hazards. In addition, volcanic activity, earthquakes, and bushfires were identified by participants from Roseau and Portsmouth. Women from Layou also identified pollution from mining as a hazard, and men from Layou identified road traffic accidents. In Layou, evacuation routes from the coast and storm drains were recognised as mitigation features. Although the vulnerability of coastal buildings was recognised by all groups, it was only in the village of Layou that mitigation measures for storm drains and shelter areas were identified.

The dream maps of all participant groups included sea walls and river embankments. However, other approaches, such as tree planting along the riverbank and seashore, creating parkland by rivers, and hazard zoning, were also called for. The need for early warning systems was recognised by most groups. There were differences between the women’s and men’s groups in Layou, with the former not only having a higher perception of the types of hazard risks but also more aspiration for developments needed to mitigate them. They were also the only group calling for the creation of more safe evacuation zones with easy access and climate-resilient housing and buildings.

## Discussion

Climate change is exposing communities in the Caribbean to some increased impacts of extreme weather and sea level rise. The region witnessed a faster rise of 3.6 mm/year in sea level than the global average (3.3 mm/year) between 1993 and 2020^89^. The impact of the rising sea level through coastal erosion and flooding, as well as the frequency of intense hurricanes accompanied by severe rainfall and high storm surges with a tendency to intrude deep into the populated areas, are likely to increase in the future^90^.

Structural intervention, financial risk transfer, contingency planning, and rapid access to recovery funds have been identified as important areas for DRR in the Caribbean region^91^. However, risk assessment is fundamental for making informed and effective decisions in these areas of DRR. Evaluation and quantification of the possible impacts through risk assessment form the basis for decision-making in different sectors such as territorial planning, resilient infrastructure, and financial protection^2^. As reiterated in multiple global DRR initiatives such as the Hyogo Framework for Action (2005–2015) and the Sendai Framework for Disaster Risk Reduction (2015–2030), a risk assessment would assist policy advice on initiating effective multipurpose mitigation measures and alleviating hazard risks in countries like Dominica.

Risk assessment studies are not easy to undertake owing to the need to be comprehensive. The investigation of multiple hazards in a single framework poses many challenges due to the different characteristics of the associated physical processes^38^. As a result, the approaches adopted in the assessments can be singular and inconsistent. Even the terminology used in dealing with multiple hazards in combinations is variable, e.g. interactions, cascades, domino effects, compound hazards, and coupled events^54^. Moreover, risk assessments that focus on multiple hazards demand extensive data for reliability, yet implementing and keeping updated large surveys of hazards, exposures, and vulnerabilities is resource-intensive. Irrespective of the approach adopted for a particular study, a precise multi-hazard risk assessment is not completely achievable. In addition, the information generation on the risk levels needs to be collected at multiple spatial scales. While the coarse-resolution data usage for investigating large areas provides generalised scenarios of risk, the assessments made through the application of simulation models and high-resolution data cover a relatively small area but with greater attention to small details.

This investigation attempted to project the spatial patterns of interconnected susceptibilities; hurricanes, being first in the hazard chain were considered an independent hazard in the present analysis, and landslides and floods the dependent ones. Here, the spatial relationship between the composite of the derived susceptibilities and demographic variables describes the scenario of hurricane risk at level-I and the application of selected models replicates the hazards at level-II.

The comprehensiveness of risk assessment studies can be improved by incorporating local expert knowledge and understanding the hazard risk perceptions of the local communities, integrated with external scientific knowledge and external risk perceptions^46,58^. The analyses here, especially using mixed methods and combining knowledge, corroborate previous work on risk assessment for Dominica—to the extent of showing large, highly localised variations and hence the need to consider multiple perspectives for effective risk assessment and management. An earlier study on risk perceptions for Dominica used a mixed methods approach where focus group participants in 18 villages produced hand-coloured maps to show where they believed volcanic risk existed on the island^13^. Parham et al.^92^ quantified the impact of educational methods for DRR by employing a longitudinal pictorial representation study of hazard perceptions from students in two secondary schools in Roseau. In these studies, risk perception was found to be highly variable, suggesting that decisions made by the people for addressing the risk at the individual or household level are uneven, and so is the state of risk.

In our study, expert interviewees and workshop participants perceived the multiple hazard risks in Dominica, some of which agreed reasonably with physical hazard mapping from this and other studies. Participants also highlighted other specific hazards, such as pollution from industry and traffic, demonstrating the breadth of hazards and risks perceived and considered. The interviewees and workshop participants also have a high perception of the aspirational measures needed to mitigate these hazard risks, corroborating the importance of including qualitative PRA tools for understanding community perception of hazards, exposures, vulnerabilities, risks, and mitigation measures. However, a high perception of risks and a willingness to act does not necessarily lead to action or other behavioural change because deeper societal factors weigh heavily in creating and continuing exposure and vulnerability^56,57,93^.

Challenges emerge when different approaches produce results that appear to diverge. Without judging which is better, more precise, or more accurate^46^, people’s perceptions may not match the modelled risks. Differing scales of assessment can make it seem as if different data and knowledge forms give conflicting results, and similarly, local views might not necessarily align. In our study, we noted some differences between workshop participants (representing mainly community views) and interviews (representing mainly local professional expertise). For instance, while workshop participants proposed in their dream maps open spaces next to rivers, engineered structural approaches in the form of sea walls and river embankments were also envisaged. By contrast, local experts did not mention focusing on or improving engineered approaches. The local expert views align with international research, which shows that the detrimental impacts arising from engineered approaches to reduce flood risk are often underestimated^94^. Similarly, disaster-resilient buildings and improving building codes and construction skills are frequently proposed as ways of DRR, but they feature little in either interview or workshop responses.

Placing our findings in the broader context of research in the eastern Caribbean, Eboh et al.^13^ found that women perceived lower overall risk than men. Our results from Layou indicate the opposite, which is more in line with the general pattern in the literature suggesting that men underestimate risk compared to women^95^. Eboh et al.^13^ also found risk denial^96^, with people who live near a volcano seeing volcanic risk as less of a threat. Again, this was not in line with our study, as we found the people of Dominica generally recognised the hazards identified by the quantitative methods and even added hazards and risks of local importance. A reason for this might be our focus on hurricane-related hazards – which aligns with recent experience and annual awareness campaigns. Perception of other hazards, namely earthquakes, volcanic eruptions, and tsunamis, would be useful for future research. Other research in the eastern Caribbean, such as Martin et al.^97^, in Trinidad found that risk perception is significantly affected by levels of income, education, and previous experience. Stancioff et al.^98^ found that residents in St. Kitts perceived negative impacts from climate and environmental change, with coastal erosion the most concerning. Yet many respondents felt more negative about society changing for the worse, including the lack of livelihood opportunities, increased crime, and worsening poverty. As with the people of Dominica noting industrial and traffic risks, it is important to consider local, social concerns when trying to raise awareness of natural hazards. The people of Dominica recognise the challenge also articulated by Shultz et al.^99,100^ that, without effective DRR, the public health consequences of Caribbean hurricane hazards would be expected to increase.

## Limitations and uncertainties

**Figure 11:**
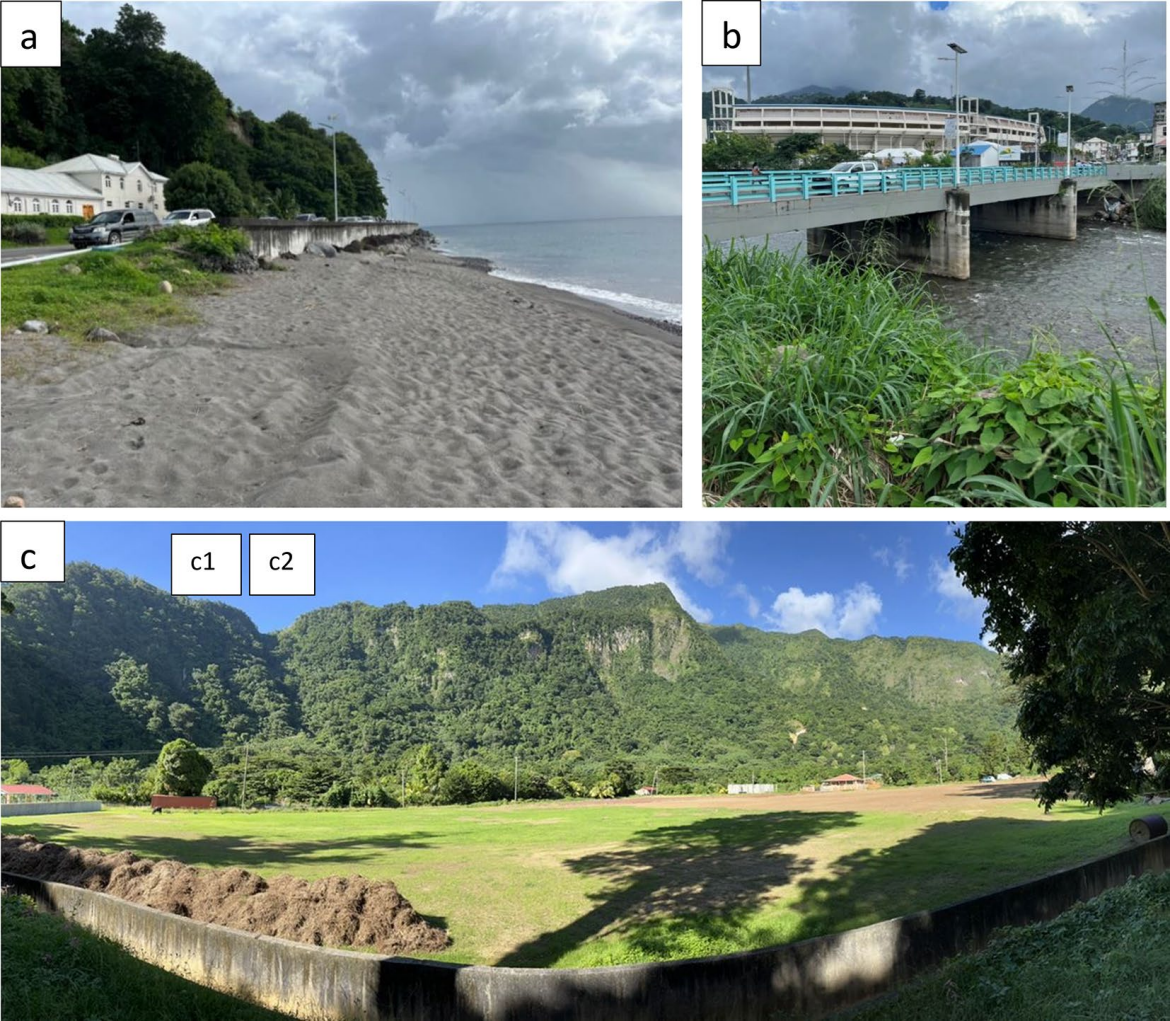
Ground validation points; (**a**) Surge simulation, location: 15° 19′ 38.4″ N 61° 23′ 42.8″ W; height of sea wall above high tide: 4.5 m, sea wall 1 m high on road side, road approximately 3.5 m above high tide mark; (**b**) bridge on Roseau River, location: 15° 18′ 07″ N 61° 23′ 10″ W, bank height on either side is approximately 5 m; (**c**) panoramic view of landslide simulation site (currently vegetated), location: 15° 14′ 13.2″ N 61° 21′ 20.0″ W, the building on extreme left is Soufriere Primary School, volume of the truncated cones at the base of the sites c1 and c2 is approximately10,000 m^3^.

Risk assessment studies involve limitations and uncertainties. For example, the parameters used for the mapping and quantification of risk are largely dynamic, changing over time and reshaping the patterns of risk. The risk parameters are in continuous flux, and their future behaviour does not necessarily remain the same as projected. For example, climate change is projected to impact the intensity and frequency of hurricanes; however, this is not explored here quantitatively. The uncertainties are also introduced from demographic and social changes, making the results of vulnerability analysis applicable for a limited period of time in the study area. Moreover, there are some constraints with regard to the quantity of data needed; vulnerability analysis is inhibited by the availability of data at the required scales. The validation of the models has been challenging mainly because of the quality of the data sets used. For instance, high-resolution LiDAR terrain data, if available could significantly improve the Level-II landslide and flood simulations results. Validating the results of RAMMS has also been difficult because there are no quantitative records (e.g. release volume and vertical and lateral spread of runout) of previous landslide events for which simulation has been done. However, ground-truthing enabled us to calculate the volume of two truncated cones at the base of the target site (Fig. 11). The variability in communities’ perception is another limitation in deriving inferences and setting DRR priorities. The challenges in themselves point to where choices of resource allocation need to be made in order to improve risk assessments and support local understandings and decisions. The limitations and uncertainties do not preclude the analyses or conclusions from having value for operational applications, as long as the they are accounted for, while further work continues to seek improvements.

## Conclusions

Dominica is repeatedly impacted by hurricanes and tropical storms. The accompanying rainfall often causes landslides and flooding; as a result, the island state experiences compound and cascading effects of multiple hazards. This study demonstrated the implementation of an integrated framework for a multi-hazard risk assessment covering hurricanes and induced landslides and floods together. The analysis was performed at two spatial scales; Level-I analysis projected and quantified multi-hazard susceptibilities and identified the at-risk population, whereas the level-II assessment produced detailed and diverse scenarios of the selected hazards at specific locations with the application of high-resolution data and simulation models. The interviews and participatory workshops provided valuable insights into how risk from various hazards is perceived and prioritised by local experts and people. The results of this analysis could be improved by interrogating more the consequence of the assumptions made for the analysis as well as including further hazards not considered here. Our research shows there is still work to be done on investigating the relevance and acceptability of hazard zoning, land use planning, building and planning regulations, and infrastructure relocation to reduce the identified risks. One possible entry point for determining the viability of these approaches is the strongly expressed community support for improving infrastructure and services. Emphasizing and enacting multi-hazard risk assessment studies, which draw on local expert and community knowledge and opinions, such as the present one, can play an important role in devising effective DRR policies and practices for Dominica and SIDS with similar challenges in the Caribbean.

### Supplementary Information

Supplementary Information.

## Data Availability

The datasets generated and/or analysed during the current study are available from the corresponding author upon reasonable request.

## References

[1] OCHA. Natural Disasters in Latin America and the Caribbean (2000–2019). https://reliefweb.int/sites/reliefweb.int/files/resources/20191203-ocha-desastres_naturales.pdf (Accessed 2 August 2021) (2020).

[2] CRED. The human cost of disasters: An overview of the last 20 years (2000–2019). https://cred.be/sites/default/files/CRED-Disaster-ReportHuman-Cost2000-2019.pdf (2020).

[3] ECLAC. Economic Commission for Latin America and the Caribbean. Financing and Planning for Disaster Risk Management in Caribbean Small Islands Developing States. https://www.cepal.org/ (Accessed 2 August 2021) (2020).

[4] World Bank. Disaster Risk Management in the Caribbean: The World Bank’s Approaches and Instruments for Recovery and Resilience, December 5, 2018 (2018).

[5] Gheuens J, Nagabhatla J, Perera EDP (2019). Disaster-risk, water security challenges and strategies in Small Island Developing States (SIDS). Water.

[6] Lindsay JM, Trumbull RB, Siebel W (2005). Geochemistry and petrogenesis of late Pleistocene to recent volcanism in Southern Dominica, Lesser Antilles. J. Volcanol. Geotherm. Res..

[7] Benson C., Clay E., Michael F. V. and Robertson A. W. Dominica: Natural, Disasters and Economic Development: in a Small Island State. Working paper series no. 2 (The World Bank, 2001).

[8] GFDRR (2015). Global Facility for Disaster Reduction and Recovery (GFDRR). Dominica—Rapid Damage and Impact Assessment: Tropical Storm Erika (English).

[9] Barclay J, Wilkinson E, White CS (2019). Historical trajectories of disaster risk in Dominica. Int. J. Disaster Risk Sci..

[10] De Graff JV, Bryce R, Jibson RW, Mora S, Rogers CT, Brabb EE, Harrod BL (1989). Landslides: Their extent and significance in the Caribbean. Landslides: Extent and Economic Significance.

[11] Charvériat C. Natural Disasters in Latin America and the Caribbean: An Overview of Risk Inter-American Development Bank, Banco Interamericano de Desarrollo (BID), Research department, Departamento de investigación, Working Paper #434 (2000).

[12] Carby B (2011). Caribbean implementation of the Hyogo Framework for Action HFA mid-term review.

[13] Eboh H, Gallaher C, Pingel T, Ashley W (2021). Risk perception in small island developing states: A case study in the Commonwealth of Dominica. Nat. Hazards.

[14] Lugo AE, Applefield M, Pool DJ, McDonald RB (1983). The impact of Hurricane David on the forests of Dominica. Can. J. For. Res..

[15] Smith RB, Schafer P, Kirshbaum D, Regina E (2009). Orographic enhancement of precipitation inside Hurricane Dean. J. Hydrometeorol..

[16] Pasch R. J., Penny A. B., and Berg R. Hurricane Maria (2017). National Hurricane Center Tropical Cyclone Report. National Hurricane Center (2019).

[17] CDERA. Summary of Impact of Hurricane “Dean" on CDERA Participating States. Response Actions, Recovery and Rehabilitation Needs Report Prepared by the Coordinating Unit of the Caribbean Disaster Emergency Response Agency (CDERA) August 22, 2007. (Accessed 22 August 2021) (2007).

[18] ACAPS. Lessons Learned – October 2017 DOMINICA. Lessons Learned from Tropical Storm Erika (2017).

[19] DMS. Rainfall data on Tropical Storm Erika, 26th to 27th August, 2015. Dominica Meteorological Service, Climate Section (2015).

[20] Kambon A., Little V., Busby L., Johnson M. and Mitchell N. The Commonwealth of Dominica: social and livelihood assessment following tropical storm Erika. Government of the Commonwealth of Dominica with the Technical Assistance of the UNDP, Barbados and the OECS (2015).

[21] PDNA. Post-Disaster Needs Assessment Hurricane Maria September 18, 2017. A Report by the Government of the Commonwealth of Dominica (2017).

[22] ACAPS. Disaster Profile: Dominica. https://reliefweb.int/sites/reliefweb.int/files/resources/20180131_acaps_disaster_profile_dominica_v2.pdf (Accessed 17 August 2021) (2018).

[23] van Westen C. and Zhang J. Tropical cyclone Maria. Inventory of landslides and flooded areas. https://unitar.org/unosat/node/44/2762 (Accessed 01 October 2021) (2018).

[24] USAID COTS Development of landslide hazard and multi-hazard assessment for Dominica, West Indies. United States Agency for International Development, Caribbean Open Trade Support Program (2016).

[25] Chiou IJ, Chen CH, Liu WL (2015). Methodology of disaster risk assessment for debris flows in a river basin. Stoch. Environ. Res. Risk Assess..

[26] Wang C, Wu J, Wang X (2018). Application of the hidden Markov model in a dynamic risk assessment of rainstorms in Dalian, China. Stoch. Environ. Res. Risk Assess..

[27] Ming X, Xu W, Li Y (2015). Quantitative multi-hazard risk assessment with vulnerability surface and hazard joint return period. Stoch. Environ. Res. Risk Assess..

[28] Kirschbaum D, Watson CS, Rounce DR, Shugar DH, Kargel JS, Haritashya UK, Amatya P, Shean D, Anderson ER, Jo M (2019). The state of remote sensing capabilities of cascading hazards over high Mountain Asia. Front. Earth Sci..

[29] Gong W, Jiang J, Yang L (2022). Dynamic risk assessment of compound hazards based on VFS–IEM–IDM: A case study of typhoon–rainstorm hazards in Shenzhen, China. Nat. Hazards Earth Syst. Sci..

[30] Crichton, D. “The risk triangle”, Natural disaster management: A presentation to commemorate the International Decade for Natural Disaster Reduction (IDNDR), pp 1990–2000 (1999).

[31] Kron W, Wu M (2002). Flood risk = hazard x exposure x vulnerability. Flood Defence.

[32] van Westen C, Kappes MS, Luna BQ, Frigerio S, Glade T, Malet J-P, van Asch T (2014). Medium-scale multi-hazard risk assessment of gravitational processes. Mountain Risks: From Prediction to Management and Governance.

[33] Granger K, Jones T, Leiba M, Scott G (1999). Community risk in Cairns: A multi-hazard risk assessment. Aust. Geol. Surv. Organ..

[34] Buck KD, Summers JK (2020). Application of a multi-hazard risk assessment for local planning. Geomat. Nat. Hazards Risk.

[35] Lung T, Lavalle C, Hiederer R, Dosio A, Bouwer LM (2013). A multi-hazard regional level impact assessment for Europe combining indicators of climatic and non-climatic change. Glob. Environ. Change.

[36] Koks EE, Rozenberg J, Zorn C (2019). A global multi-hazard risk analysis of road and railway infrastructure assets. Nat. Commun..

[37] Bell R, Glade T, Brebbia CA (2004). Multi-hazard analysis in natural risk assessments. Risk Analysis IV.

[38] Kappes MS, Keiler M, von Elverfeldt K (2012). Challenges of analyzing multi-hazard risk: A review. Nat. Hazards.

[39] Tilloy L, Malamud BD, Winter H, Joly-Laugel A (2019). A review of quantification methodologies for multi-hazard interrelationships. Earth Sci. Rev..

[40] Liu B, Siu YL, Mitchell G (2016). Hazard interaction analysis for multi-hazard risk assessment: A systematic classification based on hazard-forming environment. Nat. Hazards Earth Syst. Sci..

[41] Ming X, Liang Q, Dawson R, Xia X, Hou J (2022). A quantitative multi-hazard risk assessment framework for compound flooding considering hazard inter-dependencies and interactions. J. Hydrol..

[42] DFID. Multi-Hazard Disaster Risk Assessment (v2), UKaid. Published by the Department for International Development 2012. https://assets.publishing.service.gov.uk/ (Accessed 04 March 2023) (2012).

[43] Zhou Y, Liu Y, Wu W, Li N (2015). Integrated risk assessment of multi-hazards in China. Nat. Hazards.

[44] Hussain MA, Shuai Z, Moawwez MA, Umar T, Iqbal MR, Kamran M, Muneer MA (2023). Review of spatial variations of multiple natural hazards and risk management strategies in Pakistan. Water.

[45] Kelman I, Gaillard JC, Mercer J (2015). Climate change’s role in disaster risk reduction’s future: Beyond vulnerability and resilience. Int. J. Disaster Risk Sci..

[46] Mercer J, Kelman I, Taranis L, Suchet-Pearson S (2010). Framework for integrating indigenous and scientific knowledge for disaster risk reduction. Disasters.

[47] Chambers R (1994). The origins and practice of participatory rural appraisal. World Dev..

[48] Ahmed B, Sammonds P, Saville NM, Masson V, Le SK, Bhat GM, Hakhoo N, Jolden T, Hussain G, Wangmo K, Thusu B (2019). Indigenous mountain people’s risk perception to environmental hazards in border conflict areas. Int. J. Disaster Risk Reduct..

[49] Gill JC, Malamud BD (2016). Hazard interactions and interaction networks (cascades) within multi-hazard methodologies. Earth Syst. Dyn..

[50] Barrantes G (2018). Multi-hazard model for developing countries. Nat. Hazards.

[51] Marzocchi W, Garcia-Aristizabal A, Gasparini P, Mastellone ML, Di Ruocco A (2012). Basic principles of multi-risk assessment: A case study in Italy. Nat. Hazards.

[52] Pourghasemi HR, Kariminejad N, Amiri M (2020). Assessing and mapping multi-hazard risk susceptibility using a machine learning technique. Sci. Rep..

[53] Mignan A, Komendantova N, Scolobig A, Fleming K (2017). Multi-Risk Assessment and Governance.

[54] van Westen CJ, Greiving S (2017). Multi-hazard risk assessment and decision making. Environ. Hazards Methodol. Risk Assess. Manag..

[55] Alam A, Sammonds P, Ahmed B (2019). Cyclone risk assessment of the Cox’s Bazar and Rohingya refugee camps in southeast Bangladesh. Sci. Total Environ..

[56] Blaikie P, Cannon T, Davis I, Wisner B (1994). At Risk: Natural Hazards, Peoples’ Vulnerability and Disasters.

[57] Wisner B, Blaikie P, Cannon T, Davis I (2004). At Risk: Natural Hazards, People’s Vulnerability and Disasters.

[58] Ahmed B, Kelman I (2018). Measuring community vulnerability to environmental hazards: A method for combining quantitative and qualitative data. Nat. Hazards Rev..

[59] UNDRR. https://www.undrr.org/building-risk-knowledge/understanding-risk (Accessed 19 August 2021) (2019).

[60] Paul BK (2009). Why relatively fewer people died? The case of Bangladesh’s Cyclone Sidr. Nat. Hazards.

[61] Peduzzi P, Chatenoux B, Dao H, De Bono A, Herold C, Kossin J, Mouton F, Nordbeck O (2012). Global trends in tropical cyclone risk. Nat. Clim. Change.

[62] Smith RB, Minder JR, Nugent AD, Storelvmo T, Kirshbaum DJ, Warren R, Lareau N, Palany P, James A, French J (2012). Orographic precipitation in the tropics: The Dominica experiment. Bull. Am. Meteorol. Soc..

[63] Seo SN, Bakkensen LA (2016). Is tropical cyclone surge, not intensity, what kills so many people in south Asia?. Weather Clim. Soc..

[64] Zachry BC, Booth WJ, Rhome JR, Sharon TM (2015). A national view of storm surge risk and inundation. Weather Clim. Soc..

[65] Nugent AD, Rios-Berrios R (2018). Factors leading to extreme precipitation on Dominica from tropical storm Erika (2015). Mon. Weather Rev..

[66] Ahmed B (2015). Landslide susceptibility mapping using multi-criteria evaluation techniques in Chittagong Metropolitan Area, Bangladesh. Landslides.

[67] Ahmed B (2021). The root causes of landslide vulnerability in Bangladesh. Landslides.

[68] Lee S, Pradhan B (2007). Landslide hazard mapping at Selangor, Malaysia using frequency ratio and logistic regression models. Landslides.

[69] Sheng M, Zhou J, Chen X, Teng Y, Hong A, Liu G (2022). Landslide susceptibility prediction based on frequency ratio method and C5.0 decision tree model. Front. Earth Sci..

[70] Huang F, Yao C, Liu W, Li Y, Liu X (2018). Landslide susceptibility assessment in the Nantian area of China: A comparison of frequency ratio model and support vector machine. Geomat. Nat. Hazards Risk.

[71] Mikoš M, Bezak N (2021). Debris flow modelling using RAMMS model in the Alpine environment with focus on the model parameters and main characteristics. Front. Earth Sci..

[72] RAMMS Rapid Mass Movements Simulation (RAMMS). A numerical model for debris flows in research and practice. User Manual v1.7.0 Debris Flow (2017).

[73] Beven KJ, Kirkby MJ (1979). A physically based, variable contributing area model of basin hydrology. Hydrol. Sci. Bull..

[74] Grabs T, Seibert J, Bishop K, Laudon H (2009). Modeling spatial patterns of saturated areas: A comparison of the topographic wetness index and a dynamic distributed model. J. Hydrol..

[75] Pourali SH, Arrowsmith C, Chrisman N, Matkan AA, Mitchell D (2016). Topography wetness index application in flood-risk-based land use planning. Appl. Spat. Anal..

[76] Kelleher C, McPhillips L (2020). Exploring the application of topographic indices in urban areas as indicators of pluvial flooding locations. Hydrol. Process..

[77] Sørensen R, Zinko U, Seibert J (2006). On the calculation of the topographic wetness index: Evaluation of different methods based on field observations. Hydrol. Earth Syst. Sci..

[78] HEC-RAS. User’s Manual. https://www.hec.usace.army.mil/ (Accessed 27 May 2023) (2023).

[79] Hicks FE, Peacock T (2005). Suitability of HEC-RAS for flood forecasting. Can. Water Resour. J. Rev. Can. Ressour. Hydr..

[80] Fan C, Wang WS, Liu KFR (2012). Sensitivity analysis and water quality modeling of a Tidal river using a modified Streeter-Phelps equation with HEC-RAS-calculated hydraulic characteristics. Environ. Model. Assess..

[81] Bathrellos GD, Skilodimou HD, Chousianitis K, Youssef AM, Pradhan B (2016). Suitability estimation for urban development using multi-hazard assessment map. Sci. Total Environ..

[82] Saaty TL (2008). Decision making with the analytic hierarchy process. Int. J. Serv. Sci..

[83] Ikeda K (1995). Gender differences in human loss and vulnerability in natural disasters: A case study from Bangladesh. Indian J. Gend. Stud..

[84] Ronoh S, Gaillard JC, Marlowe J (2015). Children with disabilities and disaster risk reduction: A review. Int. J. Disaster Risk Sci..

[85] OCHA. Population Data: Facebook Connectivity Lab and Center for International Earth Science Information Network - CIESIN - Columbia University. 2016. High Resolution Settlement Layer (HRSL). Source imagery for HRSL© 2016 DigitalGlobe (2020).

[86] Heidarzadeh M, Teeuw R, Day S, Solana C (2018). Storm wave runups and sea level variations for the September 2017 Hurricane Maria along the coast of Dominica, Eastern Caribbean Sea: Evidence from field surveys and sea-level data analysis. Coast. Eng. J..

[87] Christen M, Kowalski J, Bartelt P (2010). RAMMS: Numerical simulation of dense snow avalanches in three-dimensional terrain. Cold Reg. Sci. Technol..

[88] Alam A, Ahmed B, Sammonds P (2020). Flash flood susceptibility assessment using the parameters of drainage basin morphometry in SE Bangladesh. Quat. Int..

[89] WMO. New report shows impacts of climate change and extreme weather in Latin America and Caribbean. https://public.wmo.int/ (Accessed 10 December 2021) (2021).

[90] Robinson S (2020). Climate change adaptation in SIDS: A systematic review of the literature pre and post the IPCC Fifth Assessment Report. WIREs Clim. Change.

[91] IMF. Building resilience to natural disasters in the Caribbean Requires greater preparedness by Muñoz S. and Ötker İ. https://www.imf.org/ (Accessed 14 August 2021) (2018).

[92] Parham M, Teeuw R, Solana C, Day S (2021). Quantifying the impact of educational methods for disaster risk reduction: A longitudinal study assessing the impact of teaching methods on student hazard perceptions. Int. J. Disaster Risk Reduct..

[93] Gaillard JC (2008). Alternative paradigms of volcanic risk perception: The case of Mt. Pinatubo in the Philippines. J. Volcanol. Geotherm. Res..

[94] Fordham MH (1999). The intersection of gender and social class in disaster: Balancing resilience and vulnerability. Int. J. Mass Emerg. Disasters.

[95] Finucane ML, Slovic P, Mertz CK, Flynn J, Satterfield TA (2000). Gender, race, and perceived risk: The ‘white male’ effect. Health Risk Soc..

[96] Sjöberg L (2000). Factors in risk perception. Risk Anal..

[97] Martin H, Ellis M, Delpesh C (2016). Risk perception in a multi-hazard environment: A case study of Maraval, Trinidad, The West Indian. J. Eng..

[98] Stancioff C, Stojanov R, Kelman I (2018). Local perceptions of climate change impacts in St. Kitts (Caribbean Sea) and Malé, Maldives (Indian Ocean). J. Atmos..

[99] Shultz JM, Shepherd JM, Kelman I (2018). Mitigating tropical cyclone risks and health consequences: Urgencies and innovations. Lancet Planet. Health.

[100] Shultz JM, Kossin JP, Shepherd JM (2018). Hurricane risks, health consequences, and response challenges for small island based populations: Observations from the 2017 Atlantic hurricane season. Disaster Med. Public Health Prep..

