# Author Correction: Hurricane risk assessment in a multi-hazard context for Dominica in the Caribbean

**DOI:** 10.1038/s41598-023-49300-0

**Published:** 2023-12-22

**Authors:** Peter Sammonds, Akhtar Alam, Simon Day, Katerina Stavrianaki, Ilan Kelman

**Affiliations:** 1 Institute for Risk and Disaster Reduction (IRDR), University College London (UCL), Gower Street, London, WC1E 6BT UK; 2 Department of Geography and Disaster Management, University of Kashmir, Srinagar, 190006 India; 3 Department of Statistical Science, University College London (UCL), 1‑19 Torrington Place, London, WC1E 7HB UK; 4 Institute for Global Health, University College London (UCL), Gower Street, London, WC1E 6BT UK; 5 University of Agder, Kristiansand, Norway

Correction to: *Scientific Reports* https://doi.org/10.1038/s41598-023–47527-5, Published online 23 November 2023

The original version of this Article contained an error in Figure 4 where the word “Intensity” was wrongly written as “Intensify”. The original Figure 4 and the accompanying legend appear below.

**Figure 4:**
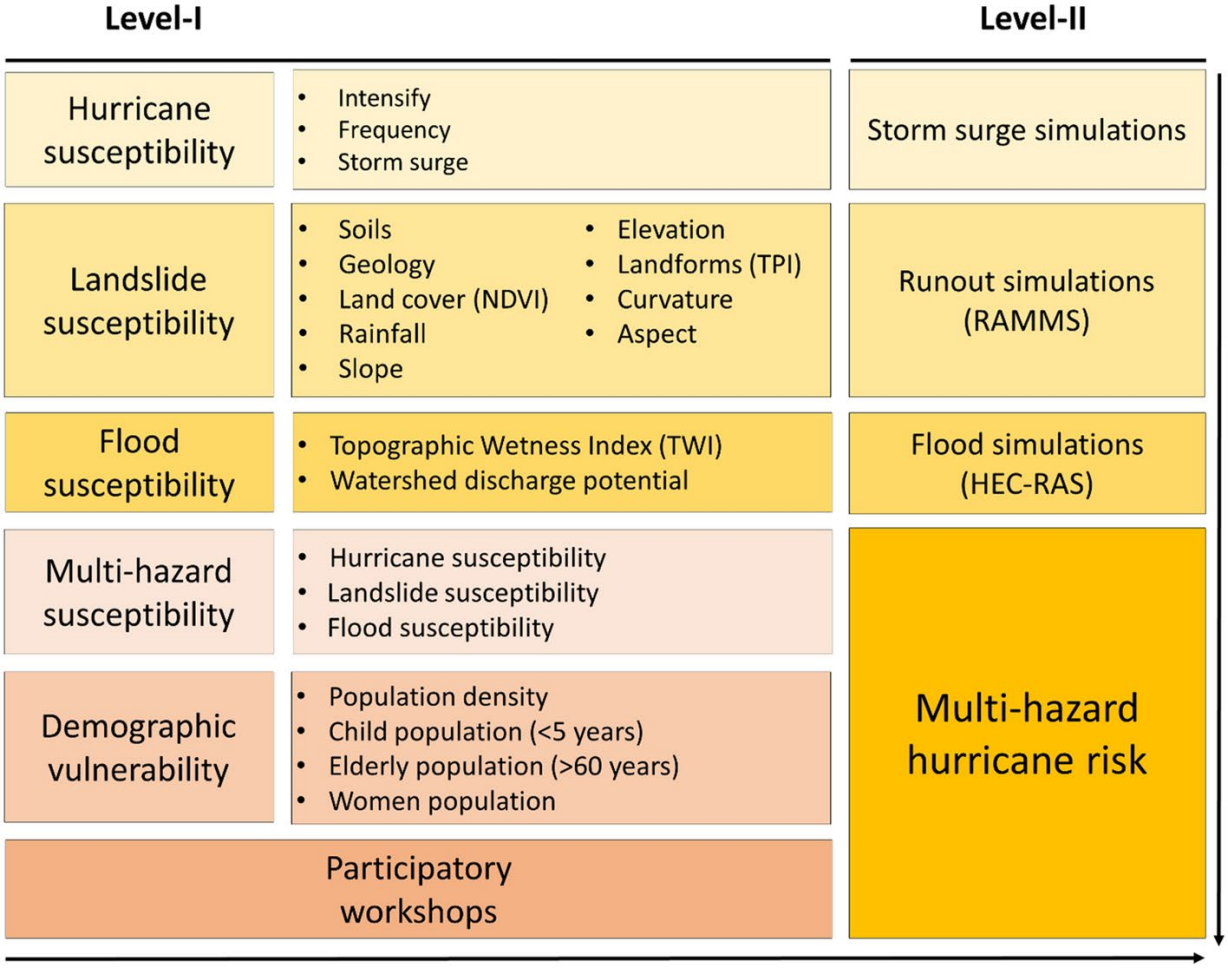
Methodological framework adopted for the present study.

The original Article has been corrected.

