# Supplementary material for: Hurricane risk assessment in a multi-hazard context for Dominica in the Caribbean
Source: Sci Rep. 2023 Nov 23;13:20565. doi: 10.1038/s41598-023-47527-5 (PMC10667232; doi:10.1038/s41598-023-47527-5)
Supplement: Supplementary file 1 — Supplementary Information. [file 41598_2023_47527_MOESM1_ESM.docx]

**Hurricane risk assessment in a multi-hazard context for Dominica in the Caribbean**

Peter Sammonds^1^, Akhtar Alam ^1,a,^*, Simon Day^1^, Katerina Stavrianaki^1,b^ and Ilan Kelman^1,c,d^

^1^Institute for Risk and Disaster Reduction (IRDR), University College London (UCL), Gower Street, London WC1E 6BT, UK.

^a^Department of Geography and Disaster Management, University of Kashmir, Srinagar, 190006, India.

^b^Department of Statistical Science, University College London (UCL), 1-19 Torrington Place, London WC1E 7HB, UK.

^c^Institute for Global Health, University College London (UCL), Gower Street, London WC1E 6BT, UK.

^d^University of Agder, Kristiansand, Norway.

* Corresponding author: [alamakhtar@uok.edu.in](mailto:alamakhtar@uok.edu.in)


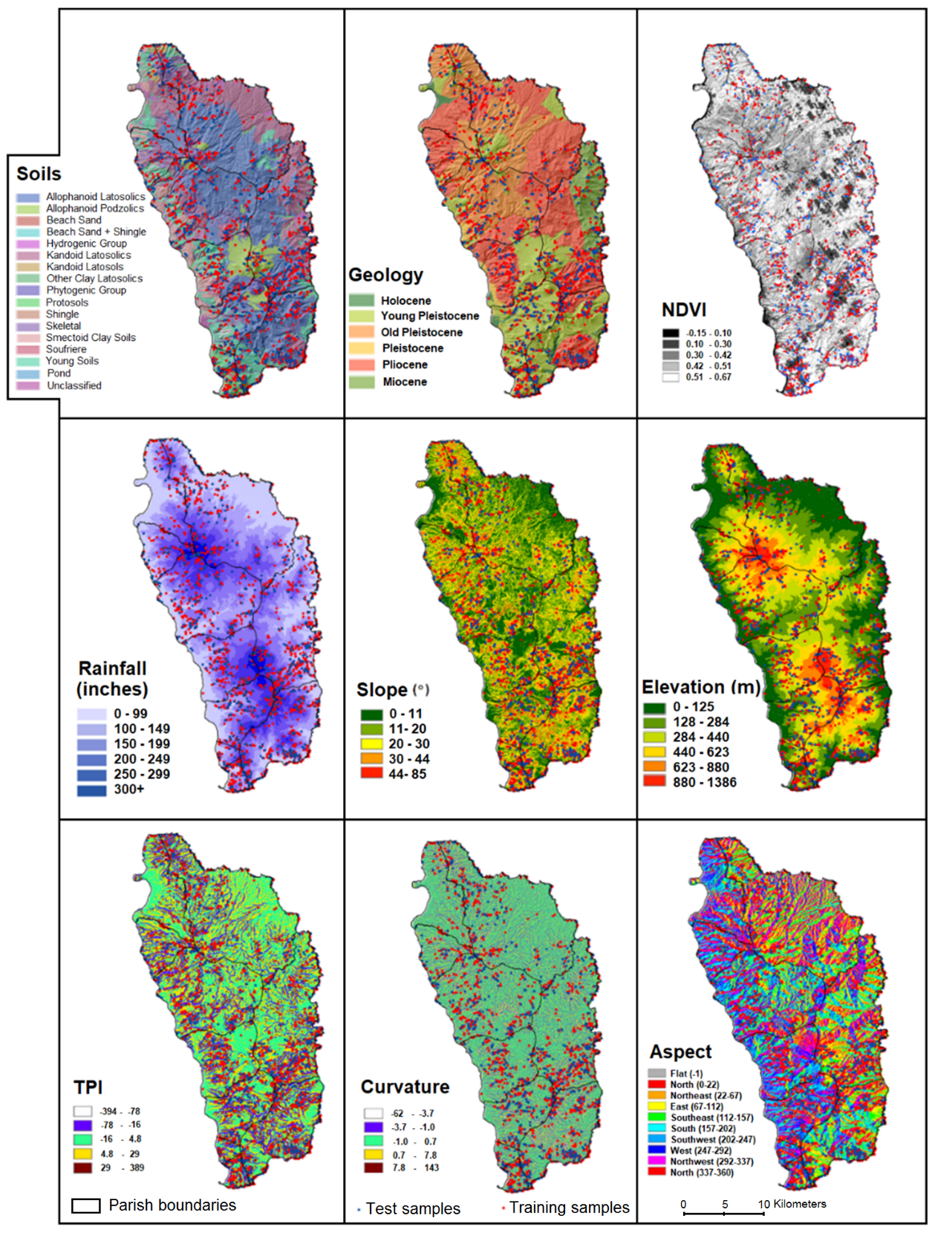


**Figure S1.** Factors selected for the landslide susceptibility analysis of Dominica. Landslide inventory shown as blue and red dots overlain on various input layers comprise of 1829 landslide events experienced from 1987 to 2020. The events (1988-2014) are from CHARIM project ([www.charim.net](http://www.charim.net)) and remaining 305 landslide events (2017 to 2020) are from the present study. NDVI: Normalized Difference Vegetation Index TPI: Topographic Position Index. Blue dots are the test landslide samples (30%) and the red dots represent training landslide samples (70%).


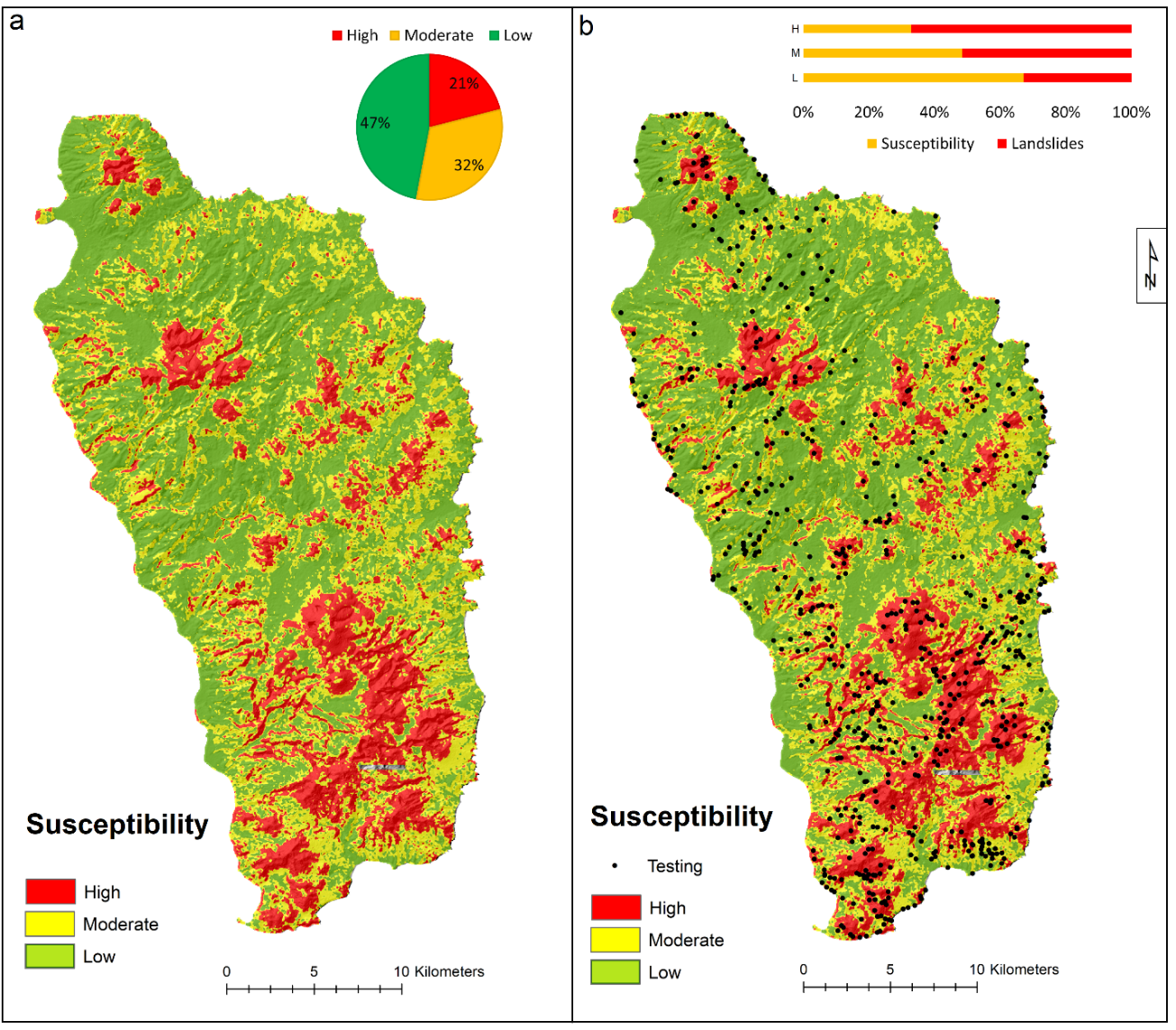


**Figure S2.** a. Landslide susceptibility map of Dominica; b. Landslide test samples (30%) shown as black dots are overlain on susceptibility map; bar graph shows correlation between the percentage of area under a particular susceptibility class and corresponding landslide frequency.


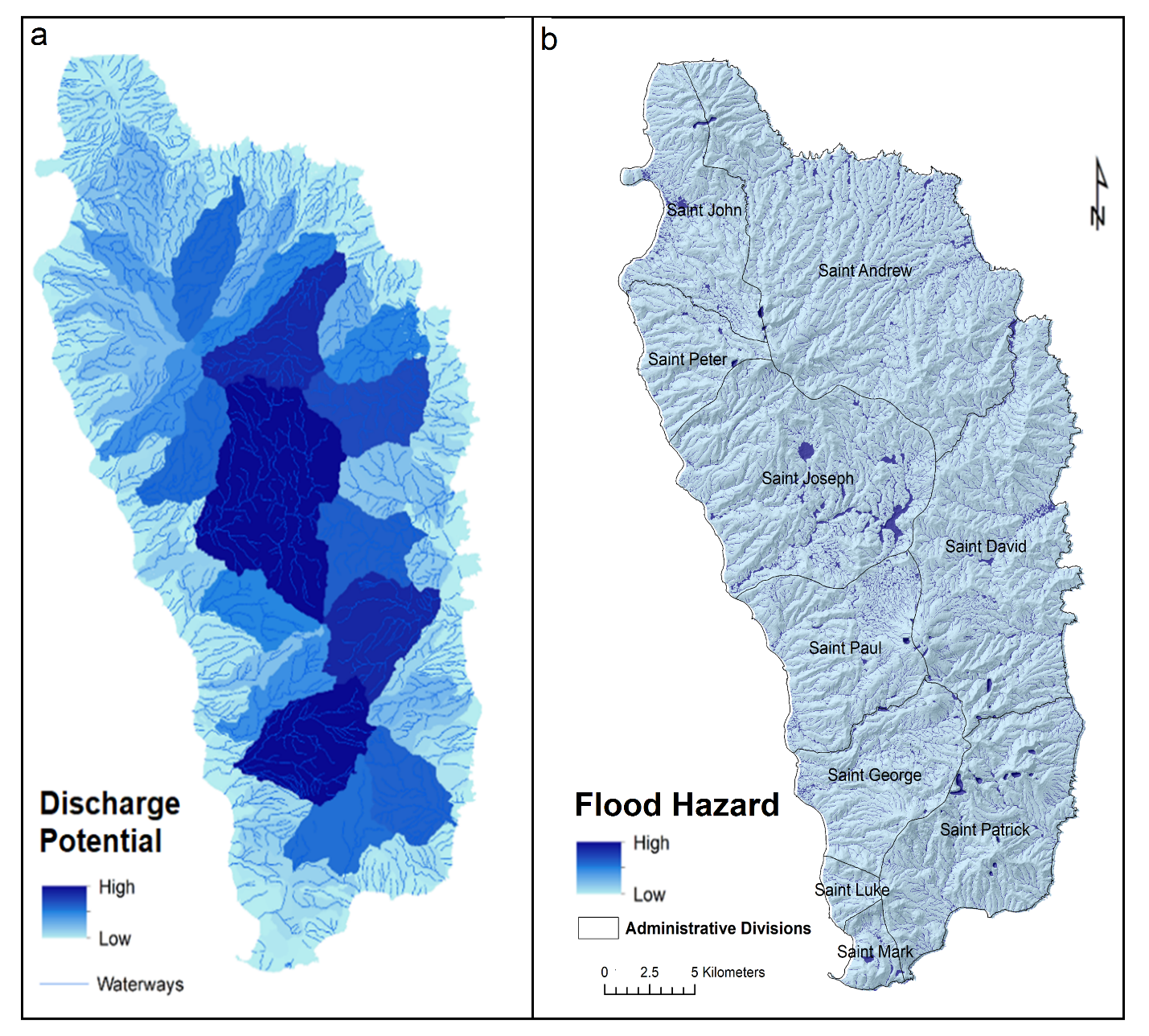


**Figure S3.** Factors selected for flood hazard analysis. a. discharge potential of the watersheds; b. Topographic Wetness Index (TWI) based flood hazard scenario.


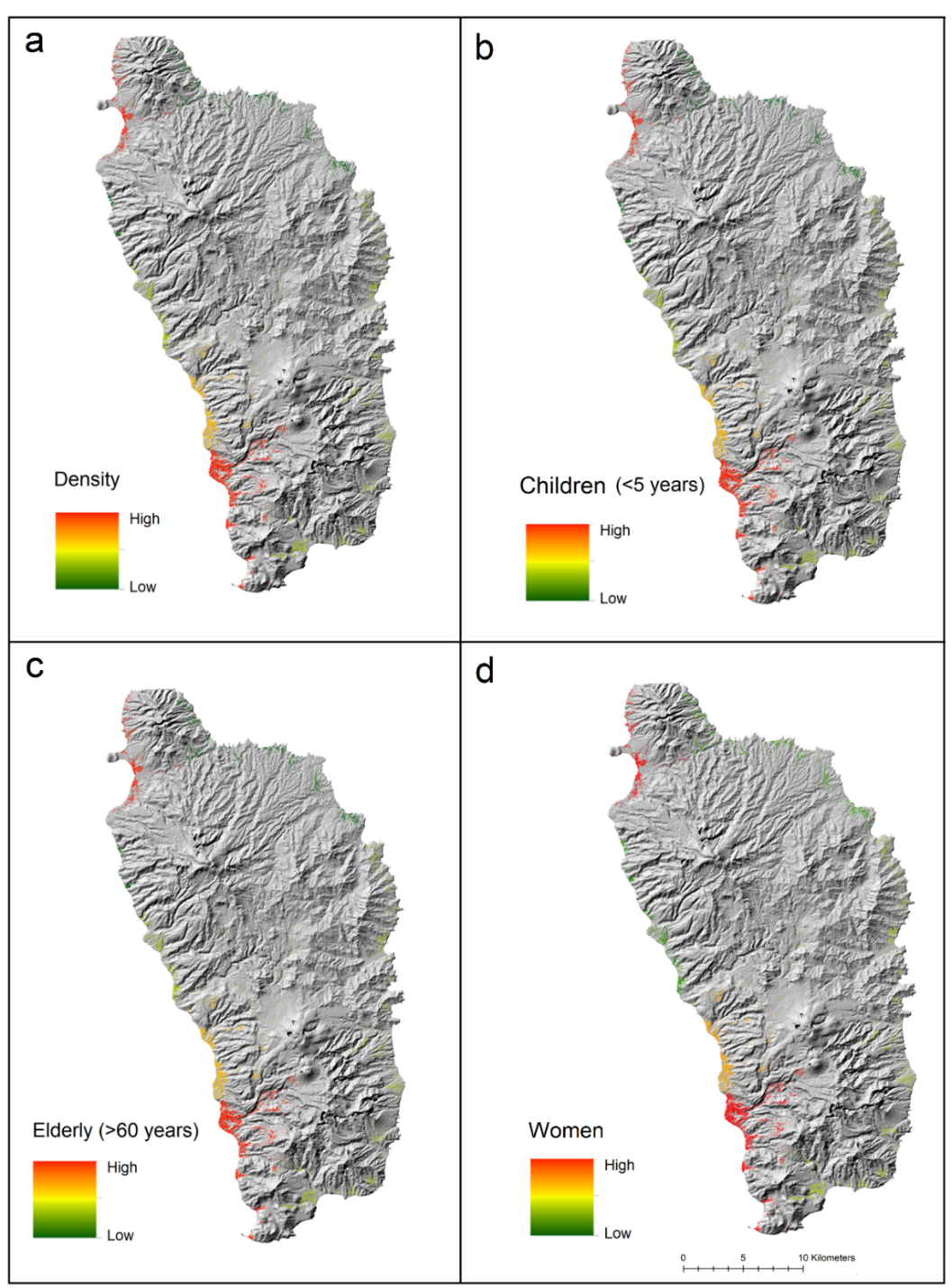


**Figure S4.** Patterns of risk sensitive demographic factors (2020); a. population density, b. children under 5 years of age, c. elderly population, d. women population.

*
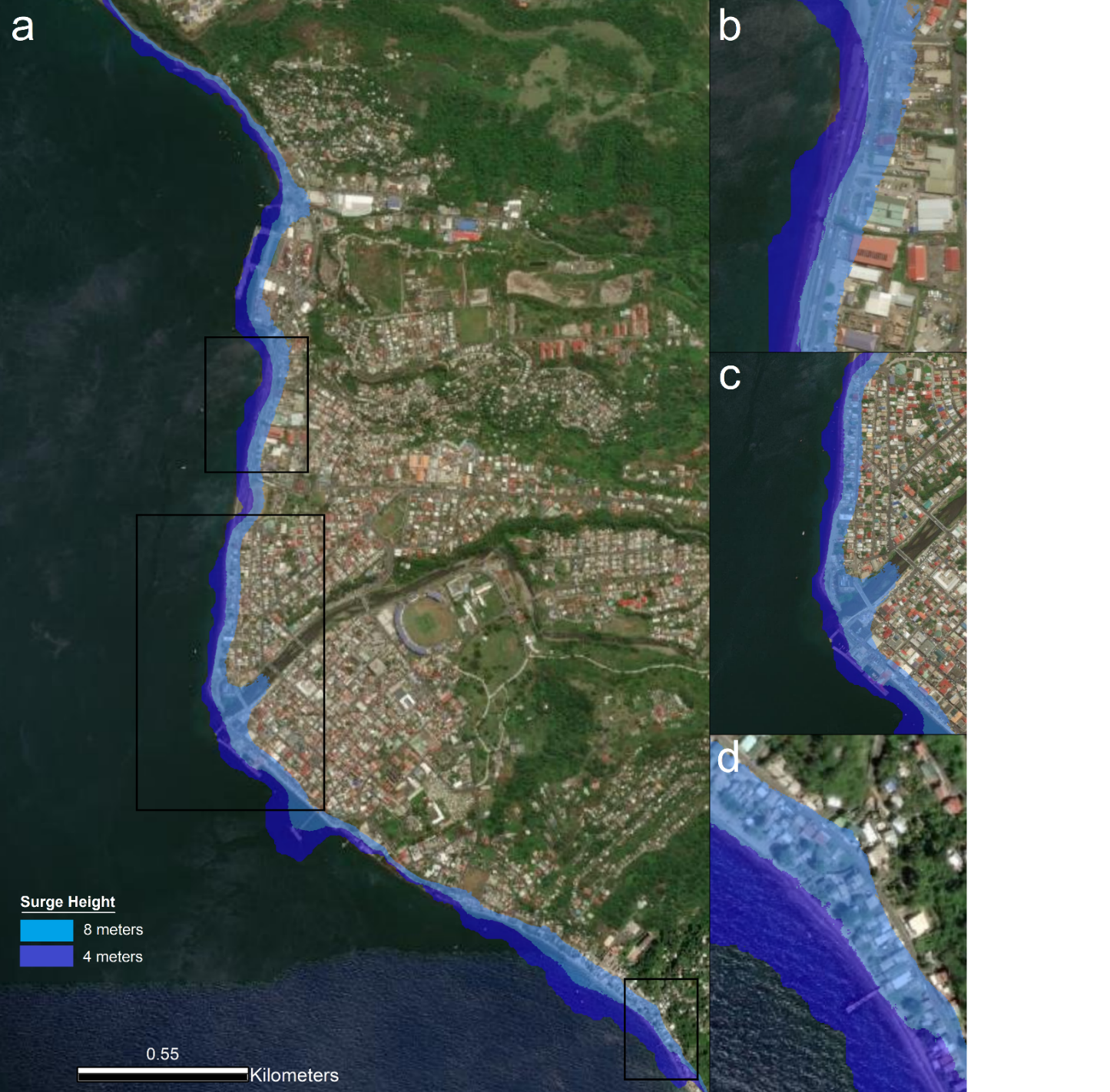
*

**Figure S5.** Storm surge scenario likely to emerge under 4m and 8m water height. The figure shows the areas that may submerge under the given surge conditions. Inset b, c and d show the zoom-in view of the locations within black colour rectangles on the ‘a’ part of the figure.


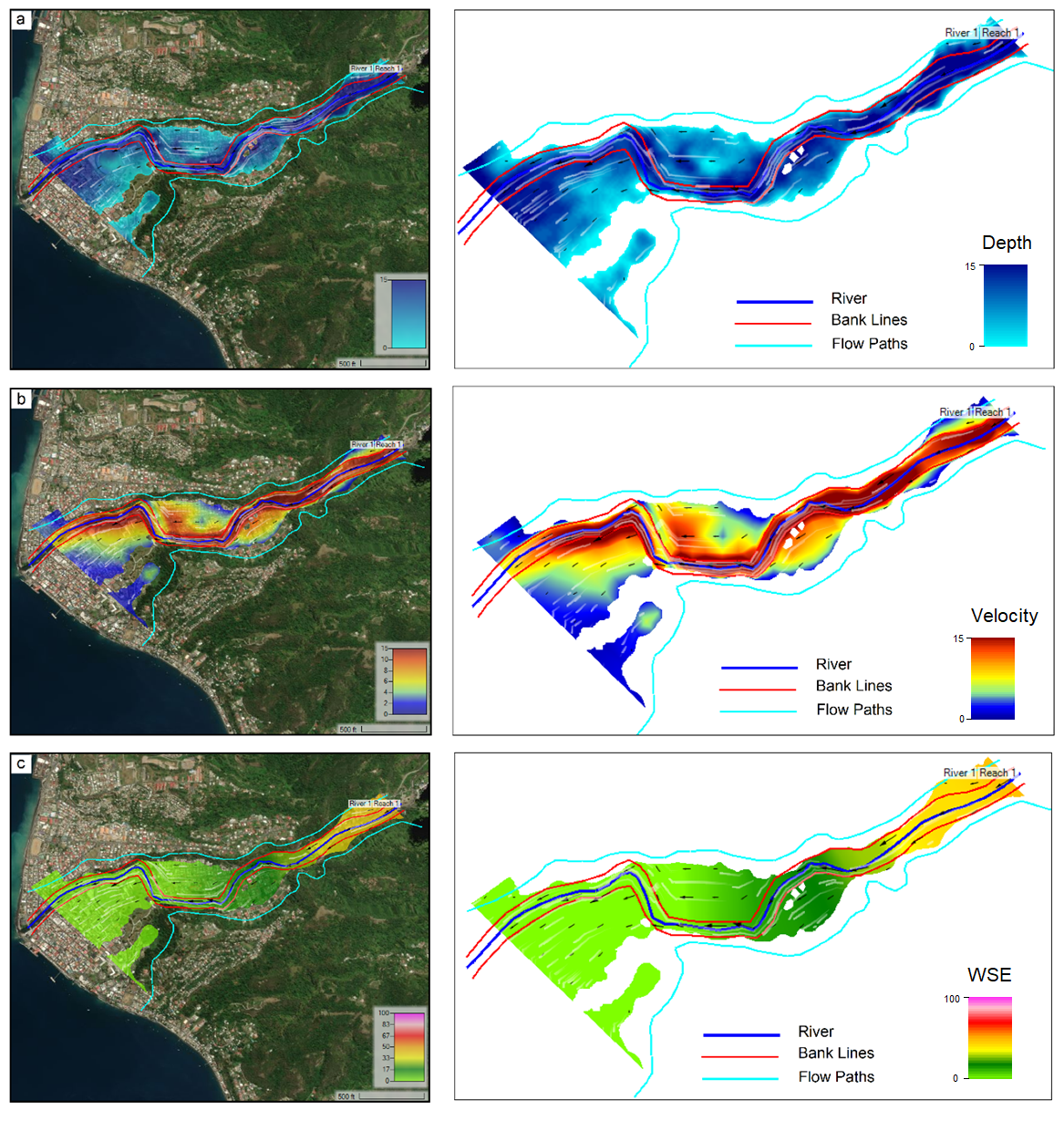


**Figure S6.** Study flow simulation of Roseau River using HEC-RAS. The scenario is developed for Q 850 (m^3^/s); a. water depth, b. velocity and c. water surface elevation (WSE) for the given discharge. Black arrows on each part show the flow direction.


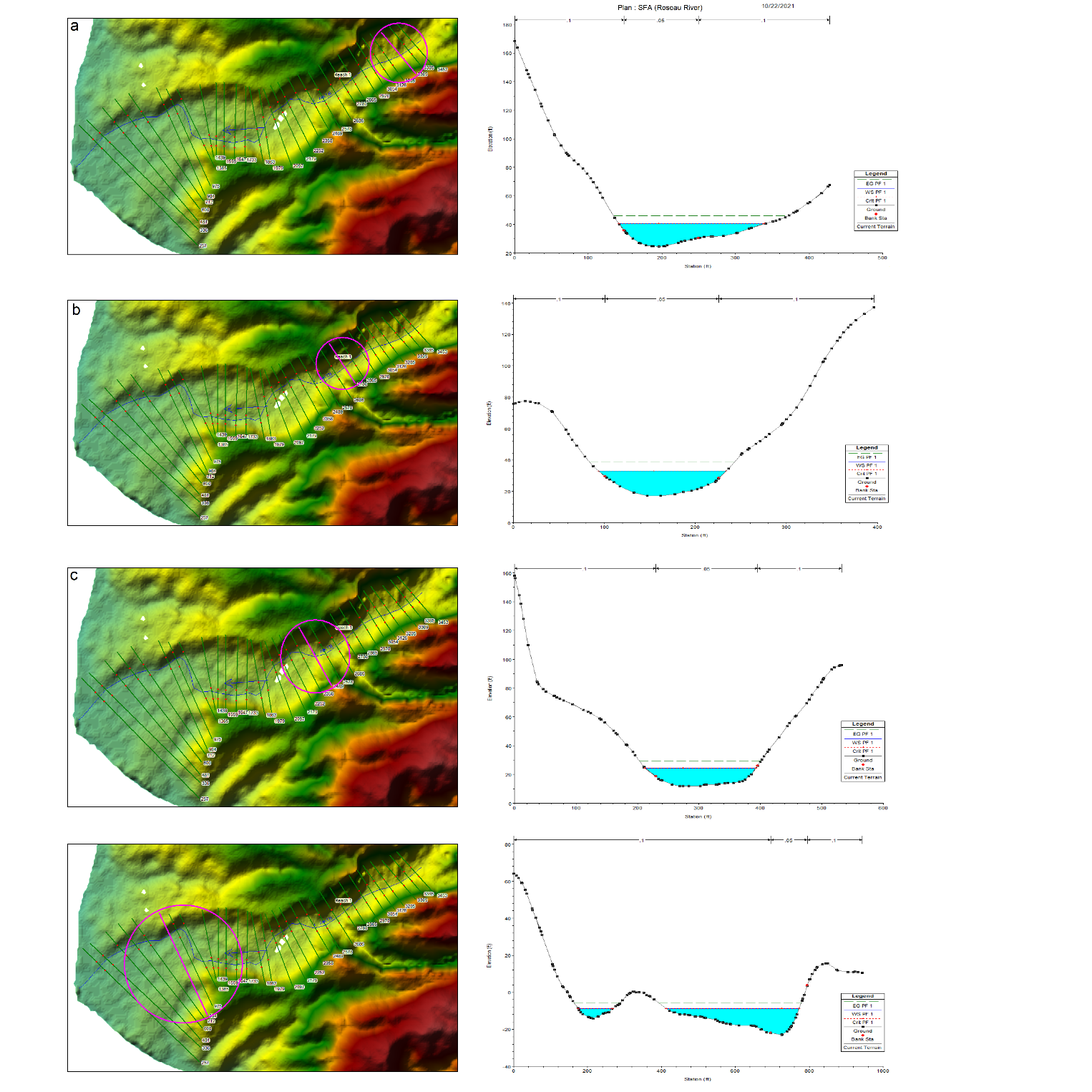


**Figure S7.** River cross section location (left) and corresponding flood water levels (right) for the given discharge.

**Table S1** Hurricanes within 60 nautical miles of Dominica (1851-2020). The intensities are based on Saffir–Simpson hurricane scale. Data has been obtained from the National Oceanic and Atmospheric Administration (NOAA) hurricane database.

| *Storm* | *Period* | | *Year* | *Maximum Category* | *Maximum Wind Speed (kt)* | *Minimum Pressure (mb)* |
| --- | --- | --- | --- | --- | --- | --- |
|  | *Start* | *End* |  |  |  |  |
| Isaias | Jul 23 | Aug 5 | 2020 | H1 | 75 | 987 |
| Dorian | Aug 24 | Sep 09 | 2019 | H5 | 160 | 910 |
| Isaac | Sep 07 | Sep15 | 2018 | H1 | 65 | 995 |
| Beryl | Jul 04 | Jul 17 | 2018 | H1 | 70 | 991 |
| Maria | Sep 16 | Oct 2 | 2017 | H5 | 150 | 908 |
| Danny | Aug 17 | Aug 24 | 2015 | H3 | 110 | 960 |
| Bertha | July 29 | Aug 09, | 2014 | H1 | 70 | 996 |
| Isaac | Aug 20 | Sep 01 | 2012 | H1 | 70 | 965 |
| Maria | Sep 06 | Sep 16 | 2011 | H1 | 70 | 983 |
| Irene | Aug 21 | Aug 30 | 2011 | H3 | 105 | 942 |
| Dean | Aug 13 | Aug 23 | 2007 | H5 | 150 | 905 |
| Jeanne | Sep 13 | Sep 29 | 2004 | H3 | 105 | 950 |
| Jose | Oct 17 | Oct 25 | 1999 | H2 | 85 | 979 |
| Hortense | Sep 03 | Sep 16 | 1996 | H4 | 120 | 935 |
| Marilyn | Sep 12 | Oct 01 | 1995 | H3 | 100 | 949 |
| Iris | Aug 22 | Sep 07 | 1995 | H2 | 95 | 957 |
| Hugo | Sep 10 | Sep 25 | 1989 | H5 | 140 | 918 |
| Gilbert | Sep 08 | Sep 20 | 1988 | H5 | 160 | 888 |
| Gert | Sep 07 | Sep15 | 1981 | H2 | 90 | 988 |
| Floyd | Sep 03 | Sep 12 | 1981 | H3 | 100 | 975 |
| Dennis | Aug 07 | Aug 22 | 1981 | H1 | 70 | 995 |
| David | Aug 25 | Sep 08 | 1979 | H5 | 150 | 924 |
| Carmen | Aug 29 | Sep 10 | 1974 | H4 | 130 | 928 |
| Holly | Sep 14 | Sep 21 | 1969 | H1 | 75 | 984 |
| Beulah | Sep 05 | Sep 22 | 1967 | H5 | 140 | 923 |
| Inez | Sep 21 | Oct11 | 1966 | H4 | 130 | 929 |
| Betsy | Aug 27 | Sep 13 | 1965 | H4 | 120 | 942 |
| Cleo | Aug 20 | Sep 11 | 1964 | H4 | 130 | 938 |
| Edith | Sep 23 | Sep 29 | 1963 | H2 | 85 | 990 |
| Frances | Sep 30 | Oct 10 | 1961 | H4 | 115 | 948 |
| Ella | Aug 30 | Sep 07 | 1958 | H2 | 95 | 983 |
| Betsy | Aug 09 | Aug 21 | 1956 | H3 | 105 | 954 |
| Charlie | Aug 12 | Aug 23 | 1951 | H4 | 115 | 968 |
| Dog | Aug 27 | Sep 05 | 1951 | H1 | 80 | 992 |
| Unnamed | Sep 20 | Sep 22 | 1949 | H1 | 70 | -1 |
| Unnamed | Aug 16 | Aug 26 | 1935 | H4 | 115 | -1 |
| Unnamed | Jul 24 | Aug 05 | 1933 | H1 | 80 | 975 |
| Unnamed | Oct 30 | Nov 14 | 1932 | H5 | 150 | 918 |
| Unnamed | Aug 29 | Sep 17 | 1930 | H4 | 135 | 933 |
| Unnamed | Sep 06 | Sep 21 | 1928 | H5 | 140 | 929 |
| Unnamed | Jul 22 | Aug 02 | 1926 | H4 | 120 | 967 |
| Unnamed | Aug 26 | Sep 06 | 1924 | H2 | 95 | 965 |
| Unnamed | Sep 02 | Sep16 | 1919 | H4 | 130 | 927 |
| Unnamed | Sep 20 | Sep 30 | 1917 | H4 | 130 | 928 |
| Unnamed | Aug 27 | Sep 02 | 1916 | H1 | 70 | 986 |
| Unnamed | Jul 10 | Jul 22 | 1916 | H2 | 90 | 980 |
| Unnamed | Aug 21 | Aug 26 | 1916 | H2 | 95 | -1 |
| Unnamed | Aug 05 | Aug 23 | 1915 | H4 | 125 | 940 |
| Unnamed | Aug 20 | Aug 28 | 1909 | H3 | 105 | -1 |
| Unnamed | Sep 21 | Oct 07 | 1908 | H2 | 95 | -1 |
| Unnamed | Aug 06 | Aug 16 | 1903 | H3 | 105 | 958 |
| Unnamed | July 04 | July 13 | 1901 | H1 | 70 | -1 |
| Unnamed | Aug 03 | Sept 04 | 1899 | H4 | 130 | 930 |
| Unnamed | Sep 05 | Sep 20 | 1898 | H2 | 95 | 965 |
| Unnamed | Sep 22 | Sep 30 | 1896 | H3 | 110 | 960 |
| Unnamed | Aug 30 | Sep 11 | 1896 | H3 | 100 | 956 |
| Unnamed | Aug 22 | Aug 30 | 1895 | H2 | 95 | -1 |
| Unnamed | Oct 11 | Oct 20 | 1894 | H4 | 115 | 931 |
| Unnamed | Sep 18 | Oct 01 | 1894 | H3 | 105 | 985 |
| Unnamed | Aug 13 | Aug 25 | 1893 | H3 | 105 | -1 |
| Unnamed | Aug 18 | Aug 25 | 1891 | H3 | 110 | 961 |
| Unnamed | Sep 12 | Sep 26 | 1889 | H2 | 95 | -1 |
| Unnamed | Sep 01 | Sep12 | 1889 | H2 | 90 | -1 |
| Unnamed | Sep 04 | Sep13 | 1883 | H3 | 110 | -1 |
| Unnamed | Sep 02 | Sep 05 | 1883 | H3 | 110 | 981 |
| Unnamed | Aug 04 | Aug 14 | 1880 | H4 | 130 | 931 |
| Unnamed | Aug 08 | Aug 19 | 1878 | H1 | 70 | -1 |
| Unnamed | Sep 08 | Sep18 | 1875 | H3 | 100 | 978 |
| Unnamed | Sep 26 | Oct 10 | 1873 | H3 | 100 | 959 |
| Unnamed | Sep 09 | Sep 20 | 1872 | H1 | 70 | -1 |
| Unnamed | Oct 08 | Oct 12 | 1867 | H1 | 75 | -1 |
| Unnamed | Sep 06 | Sep 14 | 1865 | H2 | 90 | -1 |
| Unnamed | Aug 26 | Sep 01 | 1864 | H1 | 70 | -1 |
| Unnamed | Oct 01 | Oct 16 | 1862 | H1 | 75 | 981 |
| Unnamed | Jul 06 | Jul 12 | 1861 | H2 | 90 | -1 |
| Unnamed | Aug 09 | Aug 18 | 1861 | H1 | 80 | 982 |
| Unnamed | Sep 24 | Sep 30 | 1857 | H2 | 90 | -1 |
| Unnamed | Sep 17 | Sep 30 | 1857 | H2 | 85 | -1 |
| Unnamed | Aug 16 | Aug27 | 1851 | H3 | 100 | -1 |

**Table S2** Frequency ratio statistics.

| *Factor* | *Class* | *Total pixels in a class*  *(30m, 12.5m)* | *Class pixel Frequency*  *(%)* | *Total landslide pixels in a class* | *Landslide Frequency (%)* | *Frequency Ratio (FR)* |
| --- | --- | --- | --- | --- | --- | --- |
| *Soils* | Allophanoid Latosolics | 330611 | 38.65 | 494 | 31.18 | 0.80 |
|  | Allophanoid Podzolics | 42345 | 4.95 | 50 | 3.15 | 0.68 |
|  | Beach Sand | 897 | 0.10 | 0 | 0 | 0 |
|  | Beach Sand + Shingle | 67 | 0.007 | 0 | 0 | 0 |
|  | Hydrogenic Group | 6343 | 0.74 | 7 | 0.44 | 0.59 |
|  | Kandoid latosolics | 192704 | 22.53 | 291 | 18.37 | 0.81 |
|  | Kandoid Latosols | 6084 | 0.71 | 10 | 0.63 | 0.88 |
|  | Other Clay Latosolics | 3291 | 0.38 | 0 | 0 | 0 |
|  | Phytogenic Group | 4283 | 0.50 | 1 | 0.06 | 0.12 |
|  | Protosols | 17224 | 2.01 | 7 | 0.44 | 0.21 |
|  | Shingle | 215 | 0.02 | 0 | 0 | 0 |
|  | Skeletal | 93342 | 10.91 | 420 | 26.51 | 2.42 |
|  | Smectoid Clay Soils | 41206 | 4.81 | 31 | 1.95 | 0.40 |
|  | Soufriere | 597 | 0.06 | 1 | 0.06 | 1 |
|  | Young Soils | 112495 | 13.15 | 272 | 17.17 | 1.30 |
|  | Pond | 91 | 0.01 | 0 | 0 | 0 |
|  | Unclassified | 3522 | 0.41 | 0 | 0 | 0 |
| *Geology* |  |  |  |  |  |  |
|  | Holocene | 11623 | 1.39 | 12 | 0.75 | 0.53 |
|  | Young Pleistocene | 235485 | 28.22 | 129 | 8.08 | 0.28 |
|  | Old Pleistocene | 188365 | 22.57 | 211 | 13.32 | 0.59 |
|  | Pleistocene | 8169 | 0.97 | 10 | 0.63 | 0.64 |
|  | Pliocene | 331741 | 39.75 | 633 | 39.83 | 1.00 |
|  | Miocene | 58983 | 7.06 | 593 | 37.37 | 5.29 |
| *NDVI* |  |  |  |  |  |  |
|  | -0.15 – 0.10 | 13208 | 1.58 | 26 | 2.27 | 1.43 |
|  | 0.10 – 0.30 | 71732 | 8.58 | 158 | 10.66 | 1.24 |
|  | 0.30 – 0.42 | 159557 | 19.10 | 325 | 21.27 | 1.11 |
|  | 0.42 – 0.51 | 306175 | 36.66 | 423 | 28.28 | 0.77 |
|  | 0.51 – 0.67 | 284480 | 34.06 | 584 | 37.5 | 1.10 |
| *Rainfall* |  |  |  |  |  |  |
|  | 0 - 99 | 1224352 | 25.48 | 390 | 24.62 | 0.96 |
|  | 100 - 159 | 1230140 | 25.60 | 391 | 24.68 | 0.96 |
|  | 150 - 199 | 1075518 | 22.38 | 292 | 18.43 | 0.82 |
|  | 200 - 249 | 761403 | 15.84 | 211 | 13.32 | 0.84 |
|  | 250 - 299 | 401868 | 8.36 | 228 | 14.39 | 1.72 |
|  | 300+ | 111151 | 2.31 | 72 | 4.54 | 1.96 |
| *Slope (^o^)* |  |  |  |  |  |  |
|  | 0 - 11 | 1215993 | 25.30 | 136 | 8.58 | 0.33 |
|  | 11 - 20 | 1483447 | 30.87 | 312 | 19.69 | 0.63 |
|  | 20 - 30 | 1214695 | 25.28 | 479 | 30.23 | 1.19 |
|  | 30 - 44 | 716236 | 14.90 | 492 | 31.06 | 2.08 |
|  | 44 - 85 | 174061 | 3.62 | 165 | 10.41 | 2.87 |
| *Elevation (m)* |  |  |  |  |  |  |
|  | 0 - 125 | 1224352 | 25.48 | 390 | 24.62 | 0.96 |
|  | 128 - 284 | 1230140 | 25.60 | 391 | 24.68 | 0.96 |
|  | 284 - 440 | 1075518 | 22.38 | 292 | 18.43 | 0.82 |
|  | 440 - 623 | 761403 | 15.84 | 211 | 13.32 | 0.84 |
|  | 623 - 880 | 401868 | 8.36 | 228 | 14.39 | 1.72 |
|  | 880 - 1386 | 111151 | 2.31 | 72 | 4.54 | 1.96 |
| *TPI* |  |  |  |  |  |  |
|  | -394 - -78 | 17919 | 0.37 | \| 21 \| \| --- \| | 1.32 | 3.56 |
|  | -78 - -16 | 910319 | 18.94 | 425 | 26.83 | 1.41 |
|  | -16 - 4.8 | 2060172 | 42.88 | 526 | 33.20 | 0.77 |
|  | 4.8 - 29 | 1429372 | 29.75 | 432 | 27.27 | 0.91 |
|  | 29 - 389 | 386650 | 8.04 | 180 | 11.36 | 1.41 |
|  |  |  |  |  |  |  |
| *Curvature* |  |  |  |  |  |  |
|  | -62 - -3.7 | 90500 | 1.88 | 96 | 6.06 | 3.22 |
|  | -3.7 - -1.0 | 1071311 | 22.29 | 394 | 24.87 | 1.11 |
|  | -1.0 – 0.7 | 2487537 | 51.77 | 665 | 41.98 | 0.81 |
|  | 0.7 – 7.8 | 1141871 | 23.76 | 417 | 26.32 | 1.10 |
|  | 7.8 - 143 | 13213 | 0.27 | 12 | 0.75 | 2.77 |
| *Aspect* |  |  |  |  |  |  |
|  | Flat | 11232 | 0.23 | 0 | 0 | 0 |
|  | North (0-22) | 333550 | 6.94 | 109 | 6.88 | 0.99 |
|  | Northeast (22-67) | 514955 | 10.71 | 163 | 10.29 | 0.96 |
|  | East (67-112) | 564280 | 11.74 | 193 | 12.18 | 1.03 |
|  | Southeast (112-157) | 670526 | 13.95 | 285 | 18.05 | 1.29 |
|  | South (157-202) | 618985 | 12.88 | 260 | 16.54 | 1.28 |
|  | Southwest (202-247) | 561736 | 11.69 | 162 | 10.29 | 0.88 |
|  | West (247-292) | 547334 | 11.39 | 167 | 10.54 | 0.92 |
|  | Northwest (292-337) | 659842 | 13.73 | 154 | 9.78 | 0.71 |
|  | North (337-360) | 321992 | 6.70 | 86 | 5.42 | 0.80 |

Soil, geology, NDVI=30m; Rainfall, slope, elevation, TPI, curvature, aspect=12.5m

**Table S3** Scale of relative importance (Saaty, 2008).

| *Intensity of Importance* | *Definition* | *Explanation* |
| --- | --- | --- |
| *1* | Equal Importance | Two activities contribute equally to the objective |
| *3* | Moderate importance | Experience and judgement slightly favour one activity over another |
| *5* | Strong importance | Experience and judgement strongly favour one activity over another |
| *7* | Very strong importance | An activity is favoured very strongly over another; its dominance demonstrated in practice |
| *9* | Extreme importance | The evidence favouring one activity over another is of the highest possible order of affirmation |
| *2, 4, 6 and 8* | Intermediate level of importance between numbers (1 – 3, 3 – 5, 5 – 7, and 7 – 9) |  |

**Table S4** Relative weight of the criterions derived through AHP.

|  | *H* | *L* | *F* | *Pd* | *Cp* | *Wp* | *Ep* | *Priority* | *Rank* |
| --- | --- | --- | --- | --- | --- | --- | --- | --- | --- |
| *H* | 1 | 4.00 | 5.00 | 3.00 | 2.00 | 3.00 | 2.00 | 30.3% | 1 |
| *L* | 0.25 | 1 | 3.00 | 0.33 | 0.50 | 2.00 | 1.00 | 10.2% | 4 |
| *F* | 0.20 | 0.33 | 1 | 0.33 | 0.50 | 1.00 | 1.00 | 6.4% | 7 |
| *Pd* | 0.33 | 3.00 | 3.00 | 1 | 0.25 | 1.00 | 1.00 | 12.6% | 3 |
| *Cp* | 0.50 | 2.00 | 2.00 | 4.00 | 1 | 4.00 | 3.00 | 23.8% | 2 |
| *Wp* | 0.33 | 0.50 | 1.00 | 1.00 | 0.25 | 1 | 1.00 | 7.5% | 6 |
| *Ep* | 0.50 | 1.00 | 1.00 | 1.00 | 0.33 | 1.00 | 1 | 7.5% | 5 |

*H: Hurricanes; L: Landslides; F: Floods; Pd: Population density; Cp: Children population; Wp: Women population; Ep: Elderly population*

**Table S5** Themes from the expert interviews

| *Natural hazards A* | *Infectious diseases B* | *Vulnerabilities* | *Responses* |
| --- | --- | --- | --- |
| *Hurricanes* | Covid-19 | Water access | Organisational support |
| *Landslides* | Zika | Sanitation access | Self-support |
| *Floods* | HIV | Internet access | Resilience |
| *Earthquakes* | Diseases transmitted by the *Aedes aegypti* mosquito | Infrastructure access |  |
| *Volcanic eruptions* |  | Human Resources |  |
| *Tsunami* |  | Livelihoods |  |
|  |  | Food security |  |
|  |  | Mental health |  |
|  |  | Exclusion |  |

***Appendix 1*-**Guide for Key Informant Interviews.

The following guided the interviews:

Opening discussion [to give the answers context]:

Where they work and organisational aims; who they work with; how they came to work there?

1. In your opinion, what are the main challenges facing Dominica citizens in rural and urban locations?

After the first response, then particular prompts will be given on natural hazards [hurricane, landslide, flood, earthquake, volcanic eruption, tsunami] and infectious diseases [Covid19, Zika] if they have not already mentioned.

At the: regional level [e.g. CDEMA, UWI]; national host level [political economy]; neighbourhood level; household level.

2. How do these challenges differ between different groups within Dominican society?

After the first response, then particular prompts will be given for: Men; women; male/female children; male/female elders.

3. What kind of support does your organisation offer/deliver?

a. How is that support implemented? b. How is success measured? c. How has the implementation and its success been affected by the COVID-19 pandemic? What data or information would you need to better provide support?

4. Have you observed self-support initiatives emerging? Or other non-conventional approaches?

a. In what form and led by whom? b. At what political levels? Regional, national, neighbourhood, household?

5. Does your organisation engage with these initiatives [or function in parallel to them]? What are the considerations?

6. [If not already discusses] How do you understand resilience in general? And for Dominica citizens? a. [How] does that feature in your programming?

7. Are there any additional points or questions on the themes of “multiple natural hazards” and “infectious diseases” that we have not spoken about but that we should not overlook?

Interview duration: minimum 30 minutes

***Appendix 2 -*** Themes from semi-structured interviews.

|  | *Sector* | *Hazards* | | *Vulnerabilities* | *Responses* | *Other comments* |
| --- | --- | --- | --- | --- | --- | --- |
| 1 | Education | A | Water Roads HR | |  |  |
| 2 | Fisheries | A B | Water Roads Livelihoods | | Org-support Resilience | Vaccine hesitancy |
| 3 | Health | A B | HR EDI | | Resilience | HIV Care reluctance |
| 4 | Business | A | Water Roads EDI Intranet | | Org-support Resilience | Multi-hazard |
| 5 | EDI | A | Food security HR EDI | | Org-support Resilience | GBV |
| 6 | Social services | A | Housing HR EDI Mental health | | Org-support Resilience |  |
| 7 | Agriculture | A | Food security EDI | | Org-support Resilience |  |
| 8 | Development | A | Housing EDI | | Resilience |  |
| 9 | Health | A B | Housing HR EDI | | Resilience |  |
| 10 | Health | B | EDI | |  | Mosquito control |
| 11 | Development | A B | HR EDI | | Resilience |  |
| 12 | Welfare | A B | EDI | |  |  |
| 13 | Religion | A B | Food security EDI | | Self-support |  |
| 14 | Education | A B | EDI | | Self-support |  |
| 15 | Business | A B | EDI | |  | Self-support non-evident |
| 16 | Welfare |  | Food security Roads Internet | | Self-support Resilience | Poverty, crime |
| 17 | Local government |  | Housing HR EDI | | Org-support Resilience |  |

***Appendix 3* -** Summary of participatory mapping results.

*June 28, 2021 by Dominica Red Cross*

**Layou: West Coast**

Layou Hazard Perceptions (both women’s and men’s groups)

Flooding from river

Pollution from mining and asphalt plan (women’s group only)

Storm drains mapped

Coastal surge - evacuation route (men’s group only)

Vehicular accidents (men’s group only)

Shelter/safe area (women’s group only)

**Layou Dream Map (women’s group)**

Proper wall along sea side of village (Extension of wall from village to the sea shore)

Halt all mining and dredging along river

Planting of trees along the river bank and sea shore

More storm drains

Installation of early warning system for river flooding

Creation of more safe zones with easy access

Climate resilient housing and buildings

Construction of a broad walk along the river

**Layou Dream Map (men’s group) would include:**

Construct sea defence and river walls

Halt the mining in the river and remove the asphalt plant

Construction of safety nets (gabian baskets) along the road under the cliffs

Install speed bumps in the community

Creation of a water park where the mining operations takes place and in flood zone

**Portsmouth: North-West Coast**

**Portsmouth Hazard Perceptions**

Coastal surge

Flooding due to rivers in the community and works from a construction company

Volcanic activity

**Portsmouth Dream Map**

Sea defence walls

River embankments

Construction of safety nets (gabian baskets)

Installation of signage (flood warnings, sea surge)

Creation of storm drains

**IsraAID: 12 July 2021**

**Portsmouth: North-West Coast**

Portsmouth Hazard Perceptions

Volcano

Earthquake

Bushfire

River flooding

Storm surge

Rockfalls

**Portsmouth Dream Map**

Sea defence walls

River embankments

Construction of safety nets (gabian baskets)

Creation of storm drains

Building Codes

Land use along rivers

Insurance

Frontline workers

Strengthening civil society

**Roseau: South-West Coast**

Roseau Hazard Perceptions

Volcano

Earthquake

Bushfire

River flooding

Storm surge

Rockfalls

**Roseau Dream Map**

River embankments

Construction of safety nets (gabion baskets)

Early warning system

Evacuation points

Building Codes and Land Zoning

Re-forestation

Land use along rivers

Insurance

Frontline workers

Strengthening health care
